# Repetitive Pain in Neonatal Male Rats Impairs Hippocampus-Dependent Fear Memory Later in Life

**DOI:** 10.3389/fnins.2020.00722

**Published:** 2020-07-08

**Authors:** Dongqing Xia, Cuiting Min, Yinhua Chen, Ru Ling, Mengying Chen, Xiaonan Li

**Affiliations:** Department of Child Health Care, Children’s Hospital of Nanjing Medical University, Nanjing, China

**Keywords:** procedural pain, hippocampus, fear memory, long-term potentiation, glutamate

## Abstract

Preterm infants in neonatal intensive care units are inevitably subjected to numerous painful procedures. However, little is known about the consequences of early pain experience on fear memory formation later in life. We hypothesized that exposure to repetitive pain in early life triggered hippocampal synaptic plasticity and resulted in memory deficiency in prepubertal and adult rats. From the day of birth (P0) to postnatal day 7 (P7), neonatal male rat pups were randomly assigned to either needle pricks or tactile touches repetitively every 6 h. Trace fear conditioning was performed on rats on P24–P26 and P87–P89. On P24 and P87, rats were sacrificed for molecular and electrophysiological studies. On P24–26 and P87–89, rats that experienced neonatal needle treatment showed a significant reduction in freezing time in the contextual fear conditioning (*P* < 0.05) and trace fear conditioning tests (*P* < 0.05). Moreover, repetitive neonatal procedural pain caused a significant decrease in the magnitude of hippocampal long-term potentiation induced by high-frequency stimulation. Furthermore, rats that experienced neonatal needle treatment demonstrated sustained downregulation of NR1, NR2A, NR2B, and GluR1 expression in the hippocampus. Therefore, neonatal pain is related to deficits in hippocampus-related fear memory later in life and might be caused by impairments in hippocampal synaptic plasticity.

## Introduction

Premature infants worldwide, especially those hospitalized in neonatal intensive care units (NICUs), underwent many painful procedures every day because of medical necessity (Chen et al., 2012; Cruz et al., 2016). The literature indicated that untreated pain during the critical development period might produce unfavorable neurodevelopmental outcomes, not only altering pain sensitivity but also impairing cognitive, emotional, and psychosocial function later in life (Provenzi et al., 2015; Chen et al., 2016; Walker et al., 2018).

Evidences have showed that early-life pain might lead to persistent somatosensory processing changes in both animal models and clinical studies. In young animals, basal hyperalgesia has been observed to be caused by increased input in nociceptive primary afferent axons exposed to early pain (Ruda et al., 2000; Knaepen et al., 2013; Schwaller and Fitzgerald, 2014; van den Hoogen et al., 2018), while in extremely preterm children who underwent surgery in the neonatal period, thermal sensitivity was also decreased in later life (Walker et al., 2009). Moreover, the repetitive exposure to painful procedures during the maturation of the central nervous system may certainly produce deficits in cognition in later life. Higher frequent exposure to skin-breaking procedures was associated with poorer cognition at 18 months of corrected chronological age (CCA) and even last to school age in children born very preterm (Grunau et al., 2009; Ranger et al., 2014; Vinall et al., 2014). It was also confirmed that repetitive neonatal pain in rats led to impaired spatial learning (Chen et al., 2016).

Additionally, numerous lines of evidence suggested repetitive pain occurred in neonatal period influenced the emotional development in later life. High neonatal pain experience was associated with attentional problems in toddlers born preterm (Gaspardo et al., 2018), while the rats with neonatal painful experience developed anxiety and depression-like behaviors in later life (Ranger et al., 2018; Zuke et al., 2019). However, fewer data tell us how the neonatal painful experiences influence the emotional cognition in later life, especially the fear learning.

Fear learning is a highly adaptive, evolutionarily conserved process that allows one to respond appropriately to threats in the environment. Abnormalities in fear learning are likely involved in the development and/or maintenance of neuropsychological dysfunction. Excessive fear memory is related to emotional disorders, including posttraumatic stress disorder (Chocyk et al., 2014; Villain et al., 2018); on the contrary, a lack of fear memory is usually associated with cognitive deficiency (Connor and Gould, 2016).

Pavlovian fear conditioning pairs the environment or changes in the environment (conditioned stimulus, CS) with a fearsome stimulus (unconditioned stimulus, US), providing a classical method to study fear learning. Subjects in the associative learning process rapidly learn the association between CS and US, forming the acquisition and consolidation stage of memory. After conditioning, an autonomic response to CS alone can be measured to explore memory retention. In trace fear conditioning (FC), however, a short temporal gap is placed between the CS and US, which requires additional processing by the hippocampus and prefrontal cortex (Jackson et al., 1998; Takehara et al., 2003; Yoon and Otto, 2007). Huerta found that mice with *N*-methyl-D-aspartic acid receptors (NMDARs) knocked out specifically in hippocampal CA1 pyramidal cells displayed deficits in trace FC (Huerta et al., 2000). Additionally, Kesner showed that CA1 lesions disrupted trace FC, whereas CA3 lesions did not (Hunsaker and Kesner, 2008; Hunsaker et al., 2008). These findings indicate that hippocampal CA1 pyramidal cells are crucial for trace FC.

Long-term potentiation (LTP) at synapses is widely believed to be a core mechanism of neuronal plasticity. In the hippocampus, LTP occurs, and its persistence is correlated with cognitive function (Whitlock et al., 2006). In particular, LTP in CA1 is the initial and intrinsic mechanism of memory consolidation, followed by lasting biochemical changes involving molecules such as NMDARs and α-amino-3-hydroxy-5-methyl-4-isoxazolepropionic receptors (AMPARs) to facilitate long-term maintenance of learning (Izquierdo et al., 2016; Pattwell and Bath, 2017). Interestingly, spinal LTP is also activated by C-fiber afferent activity after noxious stimulation (Ruscheweyh et al., 2011), indicating that pain perception and fear memory share some neuronal networks. Evidence showed that needle pricks in the neonatal period increased C-fiber terminal density and induced hypersensitivity of adult spinal sensory neurons (van den Hoogen et al., 2018). Likewise, neonatal tissue damage also reduced the dependence of spike-timing-dependent LTP on NMDAR activation and unmasks a novel contribution of Ca^2+^-permeable AMPARs (Li and Baccei, 2016). However, it is unknown whether neonatal pain affects hippocampal LTP, NMDARs, and AMPARs in later life.

To investigate the effects of neonatal repetitive pain during early life on fear memory in later life, we chose the needle-prick procedural pain animal model that we previously used to mimic a clinical situation (Chen et al., 2016). We examined the trace FC behavior and hippocampal synaptic plasticity through electrophysiological and molecular studies in prepubertal and adult rats that formerly experienced neonatal pain.

## Materials and Methods

### Experimental Animals

This study was performed strictly in accordance with the recommendations in the Guide for the Care and Use of Laboratory Animals of the National Institutes of Health publication 86-23. The experimental protocol was approved by the Committee on the Ethics of Animal Experiments of Nanjing Medical University (Permit Number: NJMU/IACUC2011113001). All efforts were made to minimize animal suffering, to reduce the number of animals used.

Pregnant Sprague-Dawley rats at the 14th day of pregnancy were obtained from Vital River (Beijing, China). All rats were housed individually in polycarbonate cages under a standard condition at room temperature (22 ± 3°C) under a 12/12-h light/dark cycle, with food and water available *ad libitum*.

### Experimental Design

**FIGURE 1:**
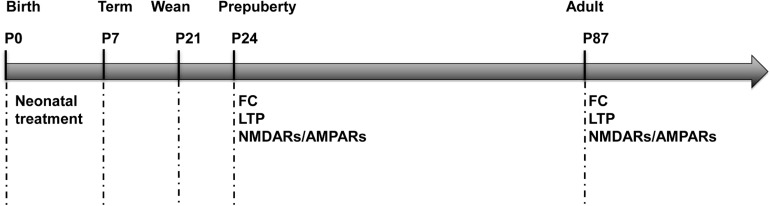
Experimental protocol. FC, fear conditioning; LTP, long-term potentiation; NMDARs, *N*-methyl-D-aspartic acid receptors; AMPARs, α-amino-3-hydroxy-5-methyl-4-isoxazolepropionic receptors.

A total of 18 pregnant SD female rats were purchased and delivered at term. On postnatal day (P) 0, the gender of rat pups was distinguished according to the ano-genital distance and only male pups (*N* = 135) were kept for future study in order to rule out the gender difference. Meanwhile, pups were standardized to *N* = 10 per dam and individual identifications were made by labeling numbers on their backs. Then they were randomly assigned into two groups, to receive different neonatal treatments from P0 to P7, either a needle prick (the needle group) or a gentle tactile stimulus (the tactile group). Litters were stayed with their mothers until weaning at P21 and after that, were housed 3 to 4 per cage. Two cohort of animals were used for the behavioral, electrophysiological and biochemical analyses on P22–26 (the tactile group: *N* = 31, the needle group: *N* = 32) and P85–89 (the tactile group: *N* = 37, the needle group: N = 35). The experimental protocol is showed in Figure 1.

Additionally, to assess the effect of repetitive tactile stimulations on behavior and gene expression, an untreated group was added as a normal control in the preliminary experiment. So a total of 48 male pups generated from additional 8 pregnant SD rats were assigned into three different groups: the untreated group, the tactile group and the needle group. Behavioral and biochemical analyses were investigated on P23–27 (*N* = 16 in the untreated, tactile and needle group, respectively). Supplementary Figure S1A provided an overview of the animals used in the preliminary study.

Each behavioral test was performed by one investigator who was blinded to the group assignment and the test was also in random order. Supplementary Figure S1B provided an overview of the animals used in the present study.

### Experimental Protocol

#### Neonatal Repetitive Procedural Treatments

To model repetitive pain exposure in the NICU, according to our previous study (Chen et al., 2016) and Anand’s study (Anand et al., 1999), pups in the needle group received four needle-prick stimulations at 6-h intervals per day from P0 to P7. Each stimulation was conducted in turns of the mid-plantar surface of the four paws by a blood glucose sampling device (Accu-Chek^®^ Softclix, Roche, Germany) set to “4,” with a sterile 28-gauge lancet (Accu-Chek^®^ Softclix, Roche, Germany). After each stimulation, the sterile lancet was replaced with a new one to prevent local inflammation. At the same time, a tactile stimulus was applied to the corresponding paws of pups in the tactile group by a cotton-tipped swab. Notably, to avoid maternal separation, the time of the pups away from their dams was controlled no more than 5 min.

In the preliminary experiment, rats in the untreated group were reared routinely without any disturbance during the neonatal period.

#### Trace Fear Conditioning Test (Trace FC)

**FIGURE 2:**
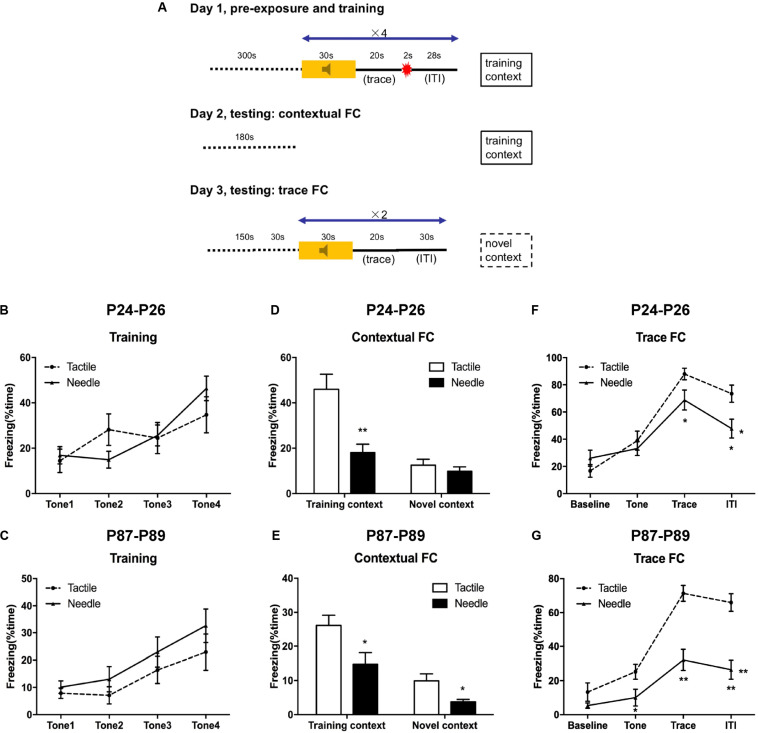
Traced fear conditioning (FC) performance. **(A)** Experimental protocol. A repeated ANOVA was used to determine the significance of differences in freezing behavior between the two groups during the training phase **(B,C)** and trace FC phase **(F,G)**. Group differences at certain time points were determined by the least significant difference (LSD) *post hoc* test. An independent *t*-test was used to detect group differences in the contextual FC phase **(D,E)**. Data are expressed as the mean ± SEM. *P < 0.05, **P < 0.01 for needle group vs. tactile group. P24: *n* (tactile group) = 12, *n* (needle group) = 15; P87: *n* = 17. ITI, intertrial interval.

Trace FC was performed on P24–P26 (the tactile group: *N* = 12, the needle group: *N* = 15) and P87–89 (*N* = 17 in the tactile and needle group, respectively) using a protocol similar to that of Anagnostaras et al.’s (1999) and Raineki et al.’s (2010) (Figure 2A). Firstly, the rats were allowed to habituate in the testing room for 3 min on 5 consecutive days prior to the beginning of the formal experiments. On the first day (training), they were trained by 4 paired training blocks after acclimating to the chamber for 5 min. Each block consisted of a 30 s, 1 kHz, 80 dB tone (CS), followed by a 20 s trace interval of silence, followed by a 2 s 0.5 mA (P24 rats) or 1.0 mA (P87 rats) foot shock (US), followed by a 28 s inter-trial interval (ITI). After each trial, the chambers were scented with a paper towel dabbed with mint solution, replaced the underneath floor and then cleaned with 75% ethanol. Freezing behavior, defined as the cessation of all but respiratory movement, was acquired by means of a time-sampling procedure using the automated motion detection software (FreezeFrame, Coulbourn Instruments, United States).

On the second day (contextual FC testing), the rats were placed in the former training chamber for 180 s to investigate the freezing behavior in the fear-conditioned context.

On the third day (trace FC testing), the rats were firstly placed in a novel chamber which was distinct from the training one, with identical parameters in the first day. In detail, prior to the tone presentation, the rats were required to acclimate the novel chamber for 150 s. If the rats showed indiscriminate freezing behavior (>50%) by the end of adaptation stage, they were excluded from further trace FC analysis. Next, the rats were presented with a 30 s baseline, followed by 2 presentations of a 30 s, 1 KHz, 80 dB tone. There was a 20 s trace interval of silence as well as a 30 s ITI between each tone presentation.

#### Electrophysiological Recordings and LTP Induction

Hippocampal LTP was recorded on P24 (the tactile group: *N* = 45 slices/9 animals, the needle group: *N* = 28 slices/7 animals) and P87 (the tactile group: *N* = 40 slices/10 animals, the needle group: *N* = 35 slices/8 animals). The rats were randomly selected from each group and decapitated for electrophysiological recording as described by Nabavi et al. (2014). The coronal hippocampal slices (350 μm) were made using a vibrating-blade microtome (Leica VT1200S) and prepared in ice-cold artificial CSF (ACSF) containing (in mM) 124 NaCl, 26 NaHCO_3_, 1.25 NaH_2_PO_4_, 2.8 KCl, 2 CaCl_2_, 2 MgSO_4_, and 10 D-glucose, oxygenated with 95% O_2_ and 5% CO_2_. Fresh slices were incubated in a chamber with carbogenated ACSF and allowed to recover at 30°C for at least 2 h.

Field excitatory postsynaptic potential (fEPSP) responses were evoked at 0.05 Hz with a 125 μm electrode placed in the middle of the stratum radiatum of CA1. A 2–3 MΩ glass recording electrode filled with 2 mM NaCl was positioned 200 μm orthodromic to the stimulating electrode. Evoked synaptic responses were elicited using 0.2–0.5 mA constant-current pulses through a metal electrode (MCE-100/200) placed in the Schaffer collateral pathway. LTP was induced by a 100 Hz high-frequency stimulation (HFS) protocol (3 trains, 100 bursts in 100 Hz with 5 s interburst intervals) at a stimulus intensity that evoked a fEPSP slope of approximately 60% of the maximum response (Nabavi et al., 2014). The averages over the last 10 min during the recording were used to calculate the magnitude of LTP.

#### Reverse Transcription Quantitative Real-Time PCR (RT-qPCR)

The rats were sacrificed by decapitation for later biochemical analyses on P24 (*N* = 10 in the tactile and needle group, respectively) and P87 (*N* = 10 in the tactile and needle group, respectively). The brains were immediately removed from the skull and the whole bilateral hippocampal tissues were quickly taken by dissection on ice. After rinsing in ice-cold saline (0.9% NaCl buffer, pH 7.4), the left and right side were stored separately in 2.0 mL microcentrifuge tubes for later Western blotting and RT-qPCR, respectively.

Total RNA was extracted from the right hippocampus homogenates using TRIzol (Invitrogen, Carlsbad, CA, United States) according to the manufacturer’s instructions. RNA quality was evaluated by a NanoDrop 1000 Spectrophotometer (Thermo, United States), while the integrity of total RNA was assessed by agarose gel electrophoresis. Total cDNA was synthesized using a Transcriptor First Strand cDNA Synthesis Kit (Roche Diagnostics GmbH, Germany) with 1.0 μg of the RNA sample, as described by the manufacturer. PCR amplification of a subset of the cDNA samples using glyceraldehyde-3-phosphate dehydrogenase (GAPDH) primers confirmed successful reverse transcription.

Real-time PCR was performed using the SYBR GREEN (Roche Diagnostics GmbH, Germany) Real-time PCR system (ABI-7500, Singapore) with the following program: 50°C for 2 min, 95°C for 10 min, 40 cycles of 95°C for 15 s and 60°C for 1 min. The mRNA levels were normalized to the corresponding GAPDH mRNA levels. Data were analyzed with the 2^–ΔΔCt^ method. The primer sequences are listed in Table 1.

**TABLE 1:** Primer sequences used for mRNA quantification by real-time PCR.

	Forward primer 5′–3′	Reverse primer 5′–3′
NR1	ACT CCC AAC GAC CAC TTC AC	ACT CGC ATC ATC TCA AAC CA
NR2A	TCC ACT CAA GGA ATC TTG TGA GAT AT	ACT TGC CCA TGT GTA TTT ATT TGT TT
NR2B	TGA TAA TGG CGG ATA AGG ATG	AGG TGG TGA CGA TGG AAA AG
GluR1	GGC TCC CTT GAC CAT AAC CT	ACG ACG CTC ACT CCA ATG TA
GAPDH	GGC TCT CTG CTC CTC CCT GTT CTA	CGT CCG ATA CGG CCA AAT CCG T

#### Western Blotting

For Western blotting, 6 left side hippocampi per group at each time point were homogenized by a tissue homogenizer (SCILOGEX D160 Homogenizer, Thomas Scientific, United States) in ice-cold lysis buffer (50 mM Tris pH 7.2, 1 mM EDTA, 0.1% SDS, 0.1% Na deoxycholate, 1% Nonidet P-40) containing a protease inhibitor cocktail (Roche). After a 30 min lysis on ice, samples were centrifuged at 13,800 × *g* for 15 min at 4°C and the supernatant was collected. Protein concentrations were determined using a Pierce bicinchoninic acid (BCA) protein assay kit with bovine serum albumin as the standard (Thermo Fisher Scientific, Rockford, IL, United States). A total of 40 μg of protein was loaded into each well of a 7.5% SDS–PAGE gel, separated and transferred onto PVDF membranes (0.45 mm; Millipore) at 4°C. Then the membranes were blocked in 5% non-fat milk for 1.5 h at room temperature, followed by incubation overnight at 4°C with a primary antibody against NR1 (1:500, Santa Cruz), NR2A (1:500, Santa Cruz), NR2B (1:500, Santa Cruz), GluR1 (1:1000, Millipore), or β-actin (1:1000, Santa Cruz). With 3 washings for 15 min each in PBS containing 0.1% TWEEN 20 (PBST), they were incubated peroxidase-conjugated goat anti-rabbit IgG secondary antibody at room temperature for 1 h (1:3000, ZSGB-BIO, China). Then washed 3 times again with PBST, blots were scanned using a ChemiDoc XRS+ Imaging System (Bio-Rad Molecular Image, United States) with electrochemiluminescence (ECL) detection reagent (Amersham, Buckinghamshire, United Kingdom). The results were expressed as the ratio of the intensity of the target protein to that of β-actin from the same membrane.

### Statistics

Statistical analysis was performed using SPSS version 16.0 for Windows (SPSS, Inc., Chicago, IL, United States). The results are presented as the mean ± SEM. For behavioral tests involving multiple trials/days per animal and body weight measurements, repeated-measures analysis of variance (ANOVA) was conducted. Group differences at certain time points were determined by independent *t*-test. Two-way ANOVA was used to detect the difference in LTP and MWT. A value of *P* < 0.05 (two-tailed) was considered statistically significant.

## Results

In the preliminary experiment, an untreated control group was added to assess the effect of repetitive tactile stimulations on behavior and gene expression. However, no differences were observed in weight gain (Supplementary Table S1), the mechanical withdrawal thresholds (Supplementary Figure S2) and fear-memory behaviors (Supplementary Figure S3) as well as the synapse-related gene expression of the hippocampi (Supplementary Figure S4) when we compared the untreated and the tactile group on P22–26. Therefore, only the tactile and needle group were set in the formal experiment to evaluate the effect of neonatal repetitive pain exposure during early life on fear memory and the underlying neurological mechanisms.

### Effects of Neonatal Pain on Later-Life Fear Conditioning in Rats

On P24–26 and P87–89, the rats were trained and tested according to protocol (Figure 2A). In the training phase, freezing behavior acquired quickly along with the consecutive tones in both group, but no differences were observed in freezing time between the needle and tactile group during the training phase, either on P24 (*F* = 0.124, *P* = 0.884, Figure 2B) or on P87 (*F* = 2.290, *P* = 0.116, Figure 2C).

Next, we observed contextual FC behavior in the training context (day 2) and the altered context (day 3). Compared to the tactile group, the needle group showed significantly reduced freezing time in the training context (*t* = 3.869, *P* = 0.001, Figure 2D) on P25, but there was no difference in the novel context (*t* = 0.836, *P* = 0.411, Figure 2D) on P26. However, the needle group exhibited reduced freezing time of contextual FC both in the training context (*t* = 2.490, *P* = 0.018) on P88 and in the novel context (*t* = 2.887, *P* = 0.007) compared to the tactile group on P89, as shown in Figure 2E.

Meanwhile, trace FC behavior in the altered context was examined on the third day. As shown in Figure 2F, the needle group exhibited a significant decrease in freezing time in the trace (*t* = 2.111, *P* = 0.045) and ITI phases (*t* = 2.668, *P* = 0.013) compared to that in the tactile group (*F* = 5.025, *P* = 0.015) on P26. Moreover, the needle group also showed a robust decrease in freezing time with trace FC (*F* = 13.091, *P* < 0.001, Figure 2G), especially during the tone (*t* = 2.282, *P* = 0.029), trace (*t* = 5.096, *P* < 0.001), and ITI phases (*t* = 5.168, *P* < 0.001) on P89. These results indicated that neonatal pain might cause memory retention deficits in later life.

### Effects of Neonatal Treatments on LTP Induced by HFS

**FIGURE 3:**
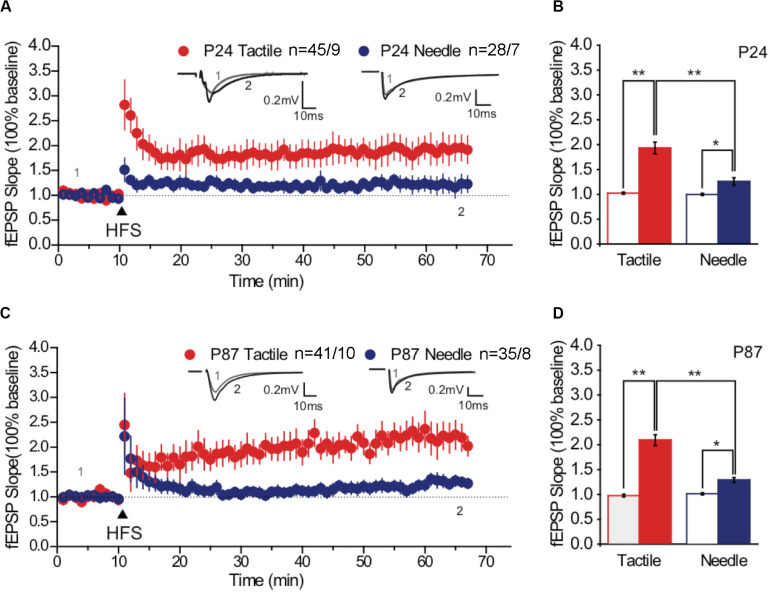
High-frequency stimulation -induced maintained LTP. Representative traces of fEPSP were obtained at baseline and after HFS stimulation (arrow) on P24 **(A)** and P87 **(C)**. Overlaid traces above the graph represented the changes in averaged fEPSPs chosen at times indicated on the graph, with the thin-lined trace representing time point 1 and the thick-lined representing time point 2. Scale bar, 0.2 mV, 10 ms. Comparative bar graph indicated the slopes of fEPSP after stimulation (solid fill), normalized to baseline (no fill) on P24 **(B)** and P87 **(D)**. Red graph represented the tactile group, while the blue graph represented the needle group. The independent t-test was used to detect group differences in the fEPSP slope. Data are expressed as the mean ± SEM. *P < 0.05, **P < 0.01 for group vs. tactile group. P24: *n* (tactile group) = 45 slices/9 rats, *n* (needle group) = 28 slices/7 rats; P87: *n* (tactile group) = 41 slices/10 rats, *n* (needle group) = 35 slices/8 rats. HFS, high-frequency stimulation; LTP, long-term potentiation.

The potentiation of the fEPSP is associated with the formation and enlargement of dendritic spines (Pickering and O’Connor, 2007). We examined the effects of neonatal treatments on synaptic plasticity by recording LTP of the hippocampal CA1 regions. Three trains of 100 pulses at 100 Hz induced LTP of fEPSPs in the CA1 neurons of both groups on P24 (Figures 3A,B) and on P87 (Figures 3C,D). According to the basal fEPSP slope, HFS-induced LTP was maintained, with the tactile and needle groups reaching 193.16 ± 11.69% and 125.73 ± 7.43% on P24 (Figure 3A) and 209.09 ± 10.67% and 128.54 ± 4.63% on P87, respectively (Figure 3C). Statistical analysis showed significant differences in group and phase both on P24 [*F*(group) = 19.122, *P* < 0.001; *F*(phase) = 53.248, *P* < 0.001] and P87 [*F*(group) = 37.280, *P* < 0.001; *F*(phase) = 123.039, *P* < 0.001]. Moreover, the intensity of LTP in the needle group was significantly lower than that in the tactile group both on P24 (*t* = 4.222, *P* < 0.001, Figure 3B) and on P87 (*t* = 6.536, *P* < 0.001, Figure 3D), indicating neonatal pain impaired hippocampal LTP from prepuberty to adulthood.

### Effects of Neonatal Pain on Synapse-Related mRNA and Protein Expression in the Hippocampus

**FIGURE 4:**
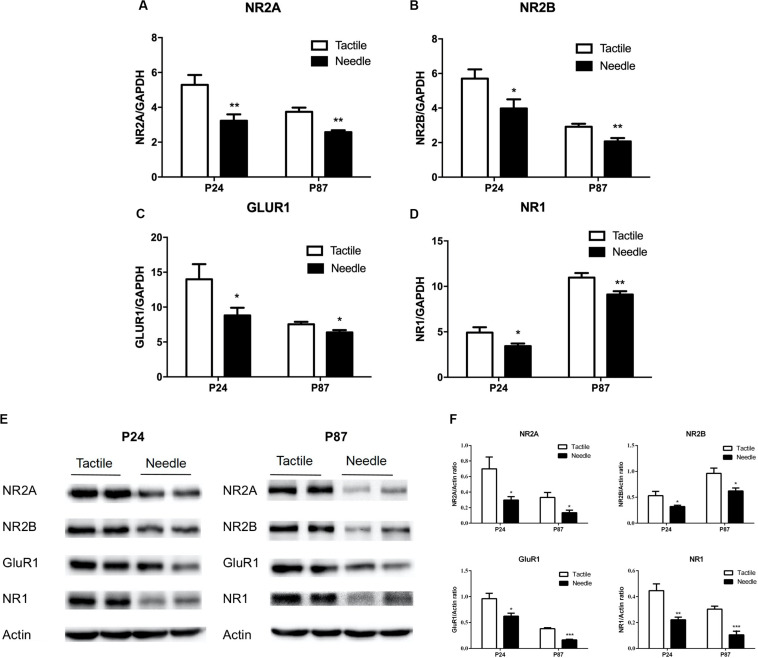
Hippocampal expression of synapse-related mRNAs and proteins. The independent *t*-test was used to detect group differences in mRNA and protein expression. The mRNA levels are presented as the ratio of each group to the control group, normalized to the corresponding GAPDH mRNA levels using the 2^– ΔΔCt^ method (*n* = 10). The relative mRNA levels of NR2A **(A)**, NR2B **(B)**, GluR1 **(C)** and NR1 **(D)** were detected on P24 and P87. **(E)** Representative blots of hippocampal synapse-related proteins and Actin. **(F)** The protein expressions of NR2A, NR2B, GluR1 and NR1 were expressed as the fold change compared with the Actin (*n* = 6). Data are expressed as the mean ± SEM. *P < 0.05, **P < 0.01 for needle group vs. tactile group.

To fully analyze the effects of neonatal pain on synaptic plasticity, we measured the mRNA and protein levels of an AMPAR subunit (GluR1) and three NMDAR subunits (NR1, NR2A, and NR2B) in whole hippocampal tissue. As shown in Figures 4A–D, mRNA levels of NR2A, NR2B, GluR1, and NR1 in the hippocampus were decreased in the needle group compared to the tactile group on both P24 (NR2A: *t* = −3.052, *P* = 0.007; NR2B: *t* = −2.305, *P* = 0.033; GluR1: *t* = −2.139, *P* = 0.046; NR1: *t* = −2.317, *P* = 0.032) and P87 (NR2A: *t* = −4.501, *P* < 0.001; NR2B: *t* = −3.208, *P* = 0.005; GluR1: *t* = −2.484, *P* = 0.023; NR1: *t* = −3.009, *P* = 0.008). Similarly, the NR2A, NR2B, GluR1, and NR1 protein expression levels were reduced in needle group rats compared with tactile group rats (P24: NR2A: *t* = −2.539, *P* = 0.029; NR2B: *t* = −2.382, *P* = 0.039; GluR1: *t* = −2.850, *P* = 0.017; NR1: *t* = −4.047, *P* = 0.002); P87: NR2A: *t* = −2.764, *P* = 0.020; NR2B: *t* = −2.245, *P* = 0.049; GluR1: *t* = −9.134, *P* < 0.001; NR1: *t* = −5.504, *P* < 0.001, shown in Figures 4E,F), indicating neonatal pain caused synapse-related molecules to change in the hippocampus.

## Discussion

Early life is a critical period of brain development due to its plasticity and vulnerability. Stressful neonatal events such as exposure to repetitive pain, light, noise, and maternal separation have long-term adverse effects on children’s brain development, including emotional and behavioral disorders. In our study, we found that neonatal pain impaired contextual and trace fear conditioning, causing sustained deficits in synaptic plasticity related to glutamate signaling from early life to adulthood. Our study expands on current knowledge regarding the impact of neonatal pain on later-life fear memory formation and its underlying mechanism.

Firstly, we demonstrated that neonatal pain had a long-term effect on fear memory. We chose both trace and contextual FC because these two types of conditioning are related to hippocampal function. Our previous study has showed that repetitive neonatal needle pricks impaired hippocampal-dependent spatial memory in the Morris water maze (Chen et al., 2016); thus, we hypothesized that the same cognitive dysfunction might occur in hippocampal emotion-related memory.

In Pavlovian cued FC, an initially non-threatening sensory stimulus, such as a tone or light, is rapidly transformed into a CS after being paired with a US that naturally evokes a defensive response (unconditioned response, such as freezing). However, CS and US information does not overlap during trace conditioning; thus, the hippocampus is engaged to bridge the interval and facilitate the formation of a CS-US association (Connor and Gould, 2016). In the present study, there was no difference between the two groups in freezing behavior during the training session, indicating that the acquisition of the CS-US association in the needle group was not affected; thus, the memory acquisition function that was dependent on the amygdala and hippocampus was not grossly impaired by neonatal stimulation.

In trace FC, there is a stimulus-free interval of several seconds – a “trace” period –between the CS and the US (Lugo et al., 2014). During this period, the CA1 of the hippocampus is believed to play an important role because lesions to the hippocampus or pharmacological agents used to block receptor function in the hippocampus result in deficits in trace conditioning and contextual conditioning (McEchron et al., 1998). Therefore, trace FC is a good method to investigate hippocampus-dependent memory that is not spatially dependent. In the present study, rats that experienced neonatal needle pricks showed impaired contextual learning behavior in the training context during prepuberty; this impairment lasted until adulthood and even increased in severity, given that the deficits occurred not only in the training context but also in the novel context in adulthood. Fear memory retrieval is tested by presenting the CS again, without the US, in a new environment. In an organism that has properly stored trace fear memory, presentation of the CS can trigger a conditioned response (CR). Our study showed that repetitive neonatal needle pricks blocked the retrieval of hippocampus-dependent trace fear memory from prepuberty to adulthood.

Until now, there have been a few published reports addressing the relationship between early-life pain and later-life fear memory. Davis’s work (Davis et al., 2018) found that needle prick produced a disruption in auditory fear conditioning when collapsed across age and sex. However, it did not show any difference in the training context and novel context. The discrepancy between our studies and Davis’s work might be due to the differences in neonatal stimulation pattern (Davis: one stimulation at an every-2-h interval only on the left hindpaw and four times per day; our study: one stimulation at an every-6-h interval on four hindpaws in turns) and in the type of fear conditioning (paradigm between cued FC and trace FC). In addition, several studies regarding early-life stress and fear memory have yielded diverse results. Chocyk et al. (2014) showed that early-life stress, modeled by maternal separation (MS), impaired fear learning during adolescence independent of sex. The effect of MS on the expression of contextual FC (both in males and in females) and auditory FC (only in males) persisted into adulthood. In our previous study, repetitive neonatal needle pricks impaired hippocampus-dependent learning and memory in the Morris water maze (Chen et al., 2016). Moreover, early-life stress impairs FC in adult rats, whereas stress experienced in adulthood has the opposite effect (Kosten et al., 2006). However, Pillai et al. (2018) found that early-life stress (induced by raising mouse pups with limited nesting and bedding material on postnatal days 2–9) did not affect context and auditory fear memory in adulthood. Therefore, there was a compelling need to examine whether neonatal pain affected fear memory later in life. More interestingly, we found that the defects in contextual FC and trace FC worsened from prepuberty to adulthood, manifested by decreased freezing behavior in contextual FC and multiple phases of trace FC.

Regarding the mechanisms of memory impairment, recent studies have suggested that LTP is crucial for memory formation (Park et al., 2016; Sachser et al., 2017). Learning involves alterations to the intrinsic excitability of hippocampal neurons and enhancement of synaptic plasticity (Disterhoft and Oh, 2006; McKay et al., 2013; Sehgal et al., 2013). There was an increase in the amplitude of synaptic potentials recorded in the hippocampus soon after contextual FC (Selig et al., 1999). Likewise, field recordings from the hippocampus of freely moving animals suggest that acquisition of FC is accompanied by a facilitation of basal synaptic transmission (Doyere et al., 1995). Specifically, mutant mice with deficient LTP in the hippocampus also exhibited deficits in contextual (but not tone) FC and trace (but not delayed) FC (Abeliovich et al., 1993; Bourtchuladze et al., 1994; Huerta et al., 2000). Therefore, LTP played an important role in facilitating contextual FC and trace FC. We found that the rats with neonatal pain experience had an impairment of maintained HFS-induced LTP at the CA1 synapses of the hippocampus both in prepuberty and adulthood. This effect might explain the deficits in fear memory in our study. Other studies reported that neonatal stress caused a reduction in hippocampal LTP (Kamal et al., 2014; Sousa et al., 2014; Lesuis et al., 2019); to our knowledge, this is the first report about the effect of neonatal pain experience on hippocampal LTP.

Pain perception and hippocampal LTP share many molecular mechanisms but also have a notable and very important distinction. A study on a different but related topic demonstrated that neonatal surgical injury relaxed the timing rules governing LTP at mouse primary afferent synapses onto mature lamina I projection neurons, which serve as a major output of the spinal nociceptive network and are essential for pain perception (Wang et al., 2019). However, it is still controversial as to whether spinal LTP and hippocampal LTP are parallel processes. For example, low-frequency afferent stimulation causes LTD at most synapses in the brain; however, low-frequency stimulation of C-fibers is sufficient to evoke LTP at a subset of dorsal horn neurons receiving direct C-fiber input (Ikeda et al., 2006). In the present study, needle pricks in the neonatal period decreased the mechanical paw withdrawal threshold, indicating hypersensitivity in somatosensory processes in later life. However, hippocampal LTP was decreased in the neonatal pain group. This may provide evidence that hippocampal LTP might not be parallel to hyperalgesia after injury. Building on this prior knowledge, our results revealed another possible instance of electrophysiological reorganization in the superior areas of the central nervous system after neonatal pain exposure.

As best characterized at the CA3–CA1 synapses of the hippocampus, conventional LTP requires calcium influx through NMDARs (Lu et al., 2001). In CA1 pyramidal cells, NMDARs contain mainly NR2A and/or NR2B subunits in addition to NR1 (Yashiro and Philpot, 2008). Glutamate signaling at NMDARs, a critical mediator of long-term memory and synaptic plasticity, is recruited within the dorsal hippocampus during both acquisition and recall of trace conditioning (Alberini, 2009). Additionally, AMPA receptors (AMPARs) play a significant role in CFC in the hippocampus. It was reported that spontaneous AMPAR-mediated synaptic transmission takes place in the hippocampal CA1 region during the association of context and shock (Zhou et al., 2009). It was demonstrated that synaptic GluR1 trafficking in the hippocampus is required for the encoding of CFC (Mitsushima et al., 2011; Takahashi, 2011). In the present study, we confirmed that neonatal repetitive procedural pain caused sustained deficits in fear memory both prepuberty and adulthood, which were associated with downregulation of NR1, NR2A, NR2B, and GluR1 in the hippocampus. In line with our finding, Blom et al. (2006) found that prenatal stress reduces the quantities of NR2B and NR2A subunits in the hippocampus of rat pups. Additionally, early postnatal chronic inflammation produces long-term changes in pain behavior and NMDAR subtype gene expression in the central nervous system of adult mice (Blom et al., 2006). Here, the reduced expression of NMDARs and an AMPAR might be the cellular mechanism for the impairment of fear memory and synaptic plasticity caused by neonatal pain.

During early postnatal development, there is a switch from slow NR2B to faster NR2A (Liu et al., 2004), as well as an increase in the ratio of AMPA to NMDA receptors (Williams et al., 1993), which is notably coinciding with the period neonatal pain occurred. This glutamate receptor switch is observed across brain regions and is crucial for synaptic maturation and cognitive development (Liu et al., 2004). Multiple studies have proved this critical process is susceptible to early life stress and glucocorticoids. Our previous major study showed neonatal repetitive pain was a powerful stressor which could activate the HPA axis even during the stress hyporesponsive period (SHRP) and disrupted the development of the HPA axis from childhood to adulthood (Chen et al., 2016). It has been suggested the hyperactivity of HPA axis disturbed the developmental mature in glutamate signaling system due to overstimulation of immature hippocampal neurons which might induce neural excitotoxicity and further contribute to the dysfunction of cognition (Popoli et al., 2011). Evidence of this propose has been observed in the rat model of maternal isolation. Mooney-Leber et al. (2018) found exposure to maternal isolation produced increase in serum corticosterone and decrease in glutamate levels, and elevated corticosterone suppressed hippocampal glutamatergic synapses and NMDA receptors (Mikasova et al., 2017).

However, in the trace FC, the different shock levels set for young and adult rats prevented the possible comparisons across the ages. Concerning the neurodevelopmental process, future studies needed to conduct under the same shock level. In addition, the duration between the shock offset and the next tone onset was only 28 s, which limited the animals with more fearful associations to the context than to the tone. Although our results indeed presented the impairments of CS-US memory retrieval in the rats experienced neonatal needle pricks, future observation are urgent to conduct under the longer ITI duration (more than 40 s). Besides, optogenetics and conditioned gene knockout in mice are promising method to clarify the causal link between neuroendocrine alternation and neonatal pain.

Overall, this study revealed that neonatal pain caused deficits in hippocampus-related fear memory and impaired hippocampal synaptic plasticity later in life, which expands the current understanding of the long-term impairment of fear memory in young and adult rats after repetitive neonatal procedural pain. The changes in synaptic plasticity and neurotransmitter metabolism in the hippocampus caused by early repeated pain stimulation in neonatal rats are important pathophysiological mechanisms for long-term behavioral development, which shed light on the significance of reducing neonatal pain in the NICU to prevent learning-process deficits in later life.

## Data Availability Statement

The original contributions presented in the study are included in the article/Supplementary Material, further inquiries can be directed to the corresponding author/s.

## Ethics Statement

The animal study was reviewed and approved by the Ethics Committee of Animal Experiments of Nanjing Medical University (Permit Number: NJMU/IACUC2011113001).

## Author Contributions

DX, MC, and CM conducted the experiments. MC and XL conceived the experiments. DX, YC, and RL analyzed the results. DX and MC wrote the manuscript. CM and XL revised the manuscript. All authors contributed to manuscript revision, read and approved the submitted version.

## Conflict of Interest

The authors declare that the research was conducted in the absence of any commercial or financial relationships that could be construed as a potential conflict of interest.

## References

[B1] Abeliovich A. Paylor R. Chen C. Kim J. J. Wehner J. M. Tonegawa S. (1993). PKC gamma mutant mice exhibit mild deficits in spatial and contextual learning. *Cell* 75 1263–1271. 10.1016/0092-8674(93)90614-v 8269510

[B2] Alberini C. M. (2009). Transcription factors in long-term memory and synaptic plasticity. *Physiol. Rev.* 89 121–145. 10.1152/physrev.00017.2008 19126756 PMC3883056

[B3] Anagnostaras S. G. Maren S. Sage J. R. Goodrich S. Fanselow M. S. (1999). Scopolamine and Pavlovian fear conditioning in rats: dose-effect analysis. *Neuropsychopharmacology* 21 731–744. 10.1016/s0893-133x(99)00083-4 10633479

[B4] Anand K. J. Coskun V. Thrivikraman K. V. Nemeroff C. B. Plotsky P. M. (1999). Long-term behavioral effects of repetitive pain in neonatal rat pups. *Physiol. Behav.* 66 627–637. 10.1016/s0031-9384(98)00338-2 10386907 PMC4211637

[B5] Blom J. M. Benatti C. Alboni S. Capone G. Ferraguti C. Brunello N. (2006). Early postnatal chronic inflammation produces long-term changes in pain behavior and N-methyl-D-aspartate receptor subtype gene expression in the central nervous system of adult mice. *J. Neurosci. Res.* 84 1789–1798. 10.1002/jnr.21077 17016858

[B6] Bourtchuladze R. Frenguelli B. Blendy J. Cioffi D. Schutz G. Silva A. J. (1994). Deficient long-term memory in mice with a targeted mutation of the cAMP-responsive element-binding protein. *Cell* 79 59–68. 10.1016/0092-8674(94)90400-6 7923378

[B7] Chaplan S. R. Bach F. W. Pogrel J. W. Chung J. M. Yaksh T. L. (1994). Quantitative assessment of tactile allodynia in the rat paw. *J. Neurosci. Methods* 53 55–63. 10.1016/0165-0270(94)90144-9 7990513

[B8] Chen M. Shi X. Chen Y. Cao Z. Cheng R. Xu Y. (2012). A prospective study of pain experience in a neonatal intensive care unit of China. *Clin. J. Pain* 28 700–704. 10.1097/ajp.0b013e3182400d54 22156826

[B9] Chen M. Xia D. Min C. Zhao X. Chen Y. Liu L. (2016). Neonatal repetitive pain in rats leads to impaired spatial learning and dysregulated hypothalamic-pituitary-adrenal axis function in later life. *Sci. Rep.* 6:39159. 10.1038/srep39159 PMC5155224 27966656

[B10] Chocyk A. Przyborowska A. Makuch W. Majcher-Maslanka I. Dudys D. Wedzony K. (2014). The effects of early-life adversity on fear memories in adolescent rats and their persistence into adulthood. *Behav. Brain Res.* 264 161–172. 10.1016/j.bbr.2014.01.040 24508235

[B11] Connor D. A. Gould T. J. (2016). The role of working memory and declarative memory in trace conditioning. *Neurobiol. Learn. Mem.* 134(Pt B), 193–209. 10.1016/j.nlm.2016.07.009 27422017 PMC5755400

[B12] Cruz M. D. Fernandes A. M. Oliveira C. R. (2016). Epidemiology of painful procedures performed in neonates: a systematic review of observational studies. *Eur. J. Pain* 20 489–498. 10.1002/ejp.757 26223408

[B13] Davis S. M. Rice M. Rudlong J. Eaton V. King T. Burman M. A. (2018). Neonatal pain and stress disrupts later-life pavlovian fear conditioning and sensory function in rats: evidence for a two-hit model. *Dev. Psychobiol.* 60 520–533. 10.1002/dev.21632 29749116 PMC6139181

[B14] Disterhoft J. F. Oh M. M. (2006). Learning, aging and intrinsic neuronal plasticity. *Trends Neurosci.* 29 587–599. 10.1016/j.tins.2006.08.005 16942805

[B15] Doyere V. Redini-Del Negro C. Dutrieux G. Le Floch G. Davis S. Laroche S. (1995). Potentiation or depression of synaptic efficacy in the dentate gyrus is determined by the relationship between the conditioned and unconditioned stimulus in a classical conditioning paradigm in rats. *Behav. Brain Res.* 70 15–29. 10.1016/0166-4328(94)00179-j 8519425

[B16] Gaspardo C. M. Cassiano R. G. M. Gracioli S. M. A. Furini G. C. B. Linhares M. B. M. (2018). Effects of neonatal pain and temperament on attention problems in toddlers born preterm. *J. Pediatr. Psychol.* 43 342–351. 10.1093/jpepsy/jsx140 29165703

[B17] Grunau R. E. Whitfield M. F. Petrie-Thomas J. Synnes A. R. Cepeda I. L. Keidar A. (2009). Neonatal pain, parenting stress and interaction, in relation to cognitive and motor development at 8 and 18 months in preterm infants. *Pain* 143 138–146. 10.1016/j.pain.2009.02.014 19307058 PMC2836793

[B18] Huerta P. T. Sun L. D. Wilson M. A. Tonegawa S. (2000). Formation of temporal memory requires NMDA receptors within CA1 pyramidal neurons. *Neuron* 25 473–480. 10.1016/s0896-6273(00)80909-5 10719900

[B19] Hunsaker M. R. Fieldsted P. M. Rosenberg J. S. Kesner R. P. (2008). Dissociating the roles of dorsal and ventral CA1 for the temporal processing of spatial locations, visual objects, and odors. *Behav. Neurosci.* 122 643–650. 10.1037/0735-7044.122.3.643 18513134

[B20] Hunsaker M. R. Kesner R. P. (2008). Dissociations across the dorsal-ventral axis of CA3 and CA1 for encoding and retrieval of contextual and auditory-cued fear. *Neurobiol. Learn. Mem.* 89 61–69. 10.1016/j.nlm.2007.08.016 17931914 PMC2675280

[B21] Ikeda H. Stark J. Fischer H. Wagner M. Drdla R. Jager T. (2006). Synaptic amplifier of inflammatory pain in the spinal dorsal horn. *Science* 312 1659–1662. 10.1126/science.1127233 16778058

[B22] Izquierdo I. Furini C. R. Myskiw J. C. (2016). Fear memory. *Physiol. Rev.* 96 695–750. 26983799 10.1152/physrev.00018.2015

[B23] Jackson P. A. Kesner R. P. Amann K. (1998). Memory for duration: role of hippocampus and medial prefrontal cortex. *Neurobiol. Learn. Mem.* 70 328–348. 10.1006/nlme.1998.3859 9774525

[B24] Kamal A. Ramakers G. M. Altinbilek B. Kas M. J. (2014). Social isolation stress reduces hippocampal long-term potentiation: effect of animal strain and involvement of glucocorticoid receptors. *Neuroscience* 256 262–270. 10.1016/j.neuroscience.2013.10.016 24161282

[B25] Knaepen L. Patijn J. Van Kleef M. Mulder M. Tibboel D. Joosten E. A. (2013). Neonatal repetitive needle pricking: plasticity of the spinal nociceptive circuit and extended postoperative pain in later life. *Dev. Neurobiol.* 73 85–97. 10.1002/dneu.22047 22821778

[B26] Kosten T. A. Lee H. J. Kim J. J. (2006). Early life stress impairs fear conditioning in adult male and female rats. *Brain Res.* 1087 142–150. 10.1016/j.brainres.2006.03.009 16626646

[B27] Lesuis S. L. Lucassen P. J. Krugers H. J. (2019). Early life stress impairs fear memory and synaptic plasticity; a potential role for GluN2B. *Neuropharmacology* 149 195–203. 10.1016/j.neuropharm.2019.01.010 30641077

[B28] Li J. Baccei M. L. (2016). Neonatal tissue damage promotes spike timing-dependent synaptic long-term potentiation in adult spinal projection neurons. *J. Neurosci.* 36 5405–5416. 10.1523/jneurosci.3547-15.2016 27170136 PMC4863065

[B29] Liu X. B. Murray K. D. Jones E. G. (2004). Switching of NMDA receptor 2A and 2B subunits at thalamic and cortical synapses during early postnatal development. *J. Neurosci.* 24 8885–8895. 10.1523/jneurosci.2476-04.2004 15470155 PMC6729956

[B30] Lu W. Man H. Ju W. Trimble W. S. Macdonald J. F. Wang Y. T. (2001). Activation of synaptic NMDA receptors induces membrane insertion of new AMPA receptors and LTP in cultured hippocampal neurons. *Neuron* 29 243–254. 10.1016/s0896-6273(01)00194-5 11182095

[B31] Lugo J. N. Smith G. D. Holley A. J. (2014). Trace fear conditioning in mice. *J. Vis. Exp.* 85:51180. 10.3791/51180 PMC4154579 24686718

[B32] McEchron M. D. Bouwmeester H. Tseng W. Weiss C. Disterhoft J. F. (1998). Hippocampectomy disrupts auditory trace fear conditioning and contextual fear conditioning in the rat. *Hippocampus* 8 638–646. 10.1002/(sici)1098-1063(1998)8:6<638::aid-hipo6>3.0.co;2-q 9882021

[B33] McKay B. M. Oh M. M. Disterhoft J. F. (2013). Learning increases intrinsic excitability of hippocampal interneurons. *J. Neurosci.* 33 5499–5506. 10.1523/jneurosci.4068-12.2013 23536065 PMC3678545

[B34] Mikasova L. Xiong H. Kerkhofs A. Bouchet D. Krugers H. J. Groc L. (2017). Stress hormone rapidly tunes synaptic NMDA receptor through membrane dynamics and mineralocorticoid signalling. *Sci. Rep.* 7:8053. 10.1038/s41598-017-08695-3 PMC5556050 28808323

[B35] Mitsushima D. Ishihara K. Sano A. Kessels H. W. Takahashi T. (2011). Contextual learning requires synaptic AMPA receptor delivery in the hippocampus. *Proc. Natl. Acad. Sci. U.S.A.* 108 12503–12508. 10.1073/pnas.1104558108 21746893 PMC3145714

[B36] Mooney-Leber S. M. Spielmann S. S. Brummelte S. (2018). Repetitive neonatal pain and reduced maternal care alter brain neurochemistry. *Dev. Psychobiol.* 60 963–974. 10.1002/dev.21777 30288732

[B37] Nabavi S. Fox R. Proulx C. D. Lin J. Y. Tsien R. Y. Malinow R. (2014). Engineering a memory with LTD and LTP. *Nature* 511 348–352. 10.1038/nature13294 24896183 PMC4210354

[B38] Park J. Chavez A. E. Mineur Y. S. Morimoto-Tomita M. Lutzu S. Kim K. S. (2016). CaMKII phosphorylation of TARPgamma-8 is a mediator of LTP and learning and memory. *Neuron* 92 75–83. 10.1016/j.neuron.2016.09.002 27667007 PMC5059846

[B39] Pattwell S. S. Bath K. G. (2017). Emotional learning, stress, and development: an ever-changing landscape shaped by early-life experience. *Neurobiol. Learn. Mem.* 143 36–48. 10.1016/j.nlm.2017.04.014 28458034 PMC5540880

[B40] Pickering M. O’Connor J. J. (2007). Pro-inflammatory cytokines and their effects in the dentate gyrus. *Prog. Brain Res.* 163 339–354. 10.1016/s0079-6123(07)63020-9 17765728

[B41] Pillai A. G. Arp M. Velzing E. Lesuis S. L. Schmidt M. V. Holsboer F. (2018). Early life stress determines the effects of glucocorticoids and stress on hippocampal function: electrophysiological and behavioral evidence respectively. *Neuropharmacology* 133 307–318. 10.1016/j.neuropharm.2018.02.001 29412144

[B42] Popoli M. Yan Z. Mcewen B. S. Sanacora G. (2011). The stressed synapse: the impact of stress and glucocorticoids on glutamate transmission. *Nat. Rev. Neurosci.* 13 22–37. 10.1038/nrn3138 22127301 PMC3645314

[B43] Provenzi L. Fumagalli M. Sirgiovanni I. Giorda R. Pozzoli U. Morandi F. (2015). Pain-related stress during the Neonatal Intensive Care Unit stay and *SLC6A4* methylation in very preterm infants. *Front. Behav. Neurosci.* 9:99. 10.3389/fnbeh.2015.00099 25941480 PMC4403508

[B44] Raineki C. Holman P. J. Debiec J. Bugg M. Beasley A. Sullivan R. M. (2010). Functional emergence of the hippocampus in context fear learning in infant rats. *Hippocampus* 20 1037–1046. 10.1002/hipo.20702 19739248 PMC2891848

[B45] Ranger M. Synnes A. R. Vinall J. Grunau R. E. (2014). Internalizing behaviours in school-age children born very preterm are predicted by neonatal pain and morphine exposure. *Eur. J. Pain* 18 844–852. 10.1002/j.1532-2149.2013.00431.x 24318537 PMC4016156

[B46] Ranger M. Tremblay S. Chau C. M. Y. Holsti L. Grunau R. E. Goldowitz D. (2018). Adverse behavioral changes in adult mice following neonatal repeated exposure to pain and sucrose. *Front. Psychol.* 9:2394. 10.3389/fpsyg.2018.02394 30719013 PMC6348336

[B47] Ruda M. A. Ling Q. D. Hohmann A. G. Peng Y. B. Tachibana T. (2000). Altered nociceptive neuronal circuits after neonatal peripheral inflammation. *Science* 289 628–631. 10915627 10.1126/science.289.5479.628

[B48] Ruscheweyh R. Wilder-Smith O. Drdla R. Liu X. G. Sandkuhler J. (2011). Long-term potentiation in spinal nociceptive pathways as a novel target for pain therapy. *Mol. Pain* 7:20. 10.1186/1744-8069-7-20 PMC3078873 21443797

[B49] Sachser R. M. Haubrich J. Lunardi P. S. De Oliveira Alvares L. (2017). Forgetting of what was once learned: exploring the role of postsynaptic ionotropic glutamate receptors on memory formation, maintenance, and decay. *Neuropharmacology* 112 94–103. 10.1016/j.neuropharm.2016.07.015 27425202

[B50] Schwaller F. Fitzgerald M. (2014). The consequences of pain in early life: injury-induced plasticity in developing pain pathways. *Eur. J. Neurosci.* 39 344–352. 10.1111/ejn.12414 24494675 PMC4264936

[B51] Sehgal M. Song C. Ehlers V. L. Moyer J. R. Jr. (2013). Learning to learn - intrinsic plasticity as a metaplasticity mechanism for memory formation. *Neurobiol. Learn. Mem.* 105 186–199. 10.1016/j.nlm.2013.07.008 23871744 PMC3855019

[B52] Selig D. K. Nicoll R. A. Malenka R. C. (1999). Hippocampal long-term potentiation preserves the fidelity of postsynaptic responses to presynaptic bursts. *J. Neurosci.* 19 1236–1246. 10.1523/jneurosci.19-04-01236.1999 9952401 PMC6786013

[B53] Sousa V. C. Vital J. Costenla A. R. Batalha V. L. Sebastiao A. M. Ribeiro J. A. (2014). Maternal separation impairs long term-potentiation in CA1-CA3 synapses and hippocampal-dependent memory in old rats. *Neurobiol. Aging* 35 1680–1685. 10.1016/j.neurobiolaging.2014.01.024 24559649

[B54] Takahashi T. (2011). Mechanisms underlying contextual fear learning. *Commun. Integr. Biol.* 4 726–727. 10.4161/cib.17505 22446538 PMC3306342

[B55] Takehara K. Kawahara S. Kirino Y. (2003). Time-dependent reorganization of the brain components underlying memory retention in trace eyeblink conditioning. *J. Neurosci.* 23 9897–9905. 10.1523/jneurosci.23-30-09897.2003 14586019 PMC6740886

[B56] van den Hoogen N. J. Patijn J. Tibboel D. Joosten B. A. Fitzgerald M. Kwok C. H. T. (2018). Repeated touch and needle-prick stimulation in the neonatal period increases the baseline mechanical sensitivity and postinjury hypersensitivity of adult spinal sensory neurons. *Pain* 159 1166–1175. 10.1097/j.pain.0000000000001201 29528964 PMC5959002

[B57] Villain H. Benkahoul A. Birmes P. Ferry B. Roullet P. (2018). Influence of early stress on memory reconsolidation: implications for post-traumatic stress disorder treatment. *PLoS One* 13:e0191563. 10.1371/journal.pone.0191563 29352277 PMC5774817

[B58] Vinall J. Miller S. P. Bjornson B. H. Fitzpatrick K. P. Poskitt K. J. Brant R. (2014). Invasive procedures in preterm children: brain and cognitive development at school age. *Pediatrics* 133 412–421. 10.1542/peds.2013-1863 24534406 PMC3934331

[B59] Walker S. M. Franck L. S. Fitzgerald M. Myles J. Stocks J. Marlow N. (2009). Long-term impact of neonatal intensive care and surgery on somatosensory perception in children born extremely preterm. *Pain* 141 79–87. 10.1016/j.pain.2008.10.012 19026489

[B60] Walker S. M. Melbourne A. O’reilly H. Beckmann J. Eaton-Rosen Z. Ourselin S. (2018). Somatosensory function and pain in extremely preterm young adults from the UK epicure cohort: sex-dependent differences and impact of neonatal surgery. *Br. J. Anaesth.* 121 623–635. 10.1016/j.bja.2018.03.035 30115261 PMC6200114

[B61] Wang X.-M. Pan W. Xu N. Zhou Z.-Q. Zhang G.-F. Shen J.-C. (2019). Environmental enrichment improves long-term memory impairment and aberrant synaptic plasticity by BDNF/TrkB signaling in nerve-injured mice. *Neurosci. Lett.* 694 93–98. 10.1016/j.neulet.2018.11.049 30496785

[B62] Whitlock J. R. Heynen A. J. Shuler M. G. Bear M. F. (2006). Learning induces long-term potentiation in the hippocampus. *Science* 313 1093–1097. 10.1126/science.1128134 16931756

[B63] Williams K. Russell S. L. Shen Y. M. Molinoff P. B. (1993). Developmental switch in the expression of NMDA receptors occurs in vivo and in vitro. *Neuron* 10 267–278. 10.1016/0896-6273(93)90317-k 8439412

[B64] Yashiro K. Philpot B. D. (2008). Regulation of NMDA receptor subunit expression and its implications for LTD, LTP, and metaplasticity. *Neuropharmacology* 55 1081–1094. 10.1016/j.neuropharm.2008.07.046 18755202 PMC2590778

[B65] Yoon T. Otto T. (2007). Differential contributions of dorsal vs. ventral hippocampus to auditory trace fear conditioning. *Neurobiol. Learn. Mem.* 87 464–475. 10.1016/j.nlm.2006.12.006 17251041

[B66] Zhou M. Conboy L. Sandi C. Joels M. Krugers H. J. (2009). Fear conditioning enhances spontaneous AMPA receptor-mediated synaptic transmission in mouse hippocampal CA1 area. *Eur. J. Neurosci.* 30 1559–1564. 10.1111/j.1460-9568.2009.06951.x 19811531

[B67] Zuke J. T. Rice M. Rudlong J. Paquin T. Russo E. Burman M. A. (2019). The effects of acute neonatal pain on expression of corticotropin-releasing hormone and juvenile anxiety in a rodent model. *eNeuro* 6:ENEURO.0162-19.2019. 10.1523/ENEURO.0162-19.2019 PMC6860982 31601633

