# Supplementary figures and images for: Repetitive Pain in Neonatal Male Rats Impairs Hippocampus-Dependent Fear Memory Later in Life
Source: Front Neurosci. 2020 Jul 8;14:722. doi: 10.3389/fnins.2020.00722 (PMC7360690; doi:10.3389/fnins.2020.00722)

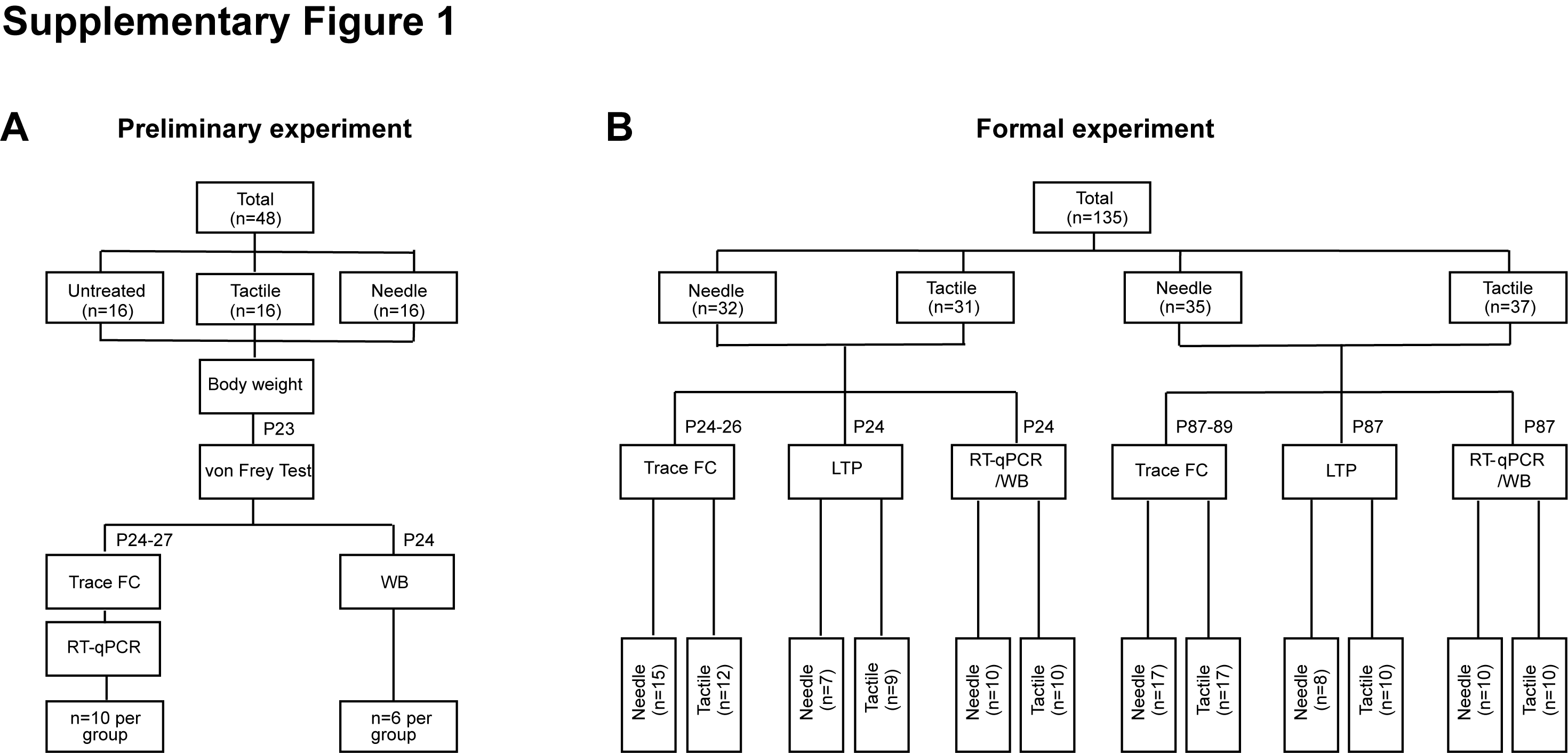

Supplement: Supplementary file 1 [file Image_1.tif]

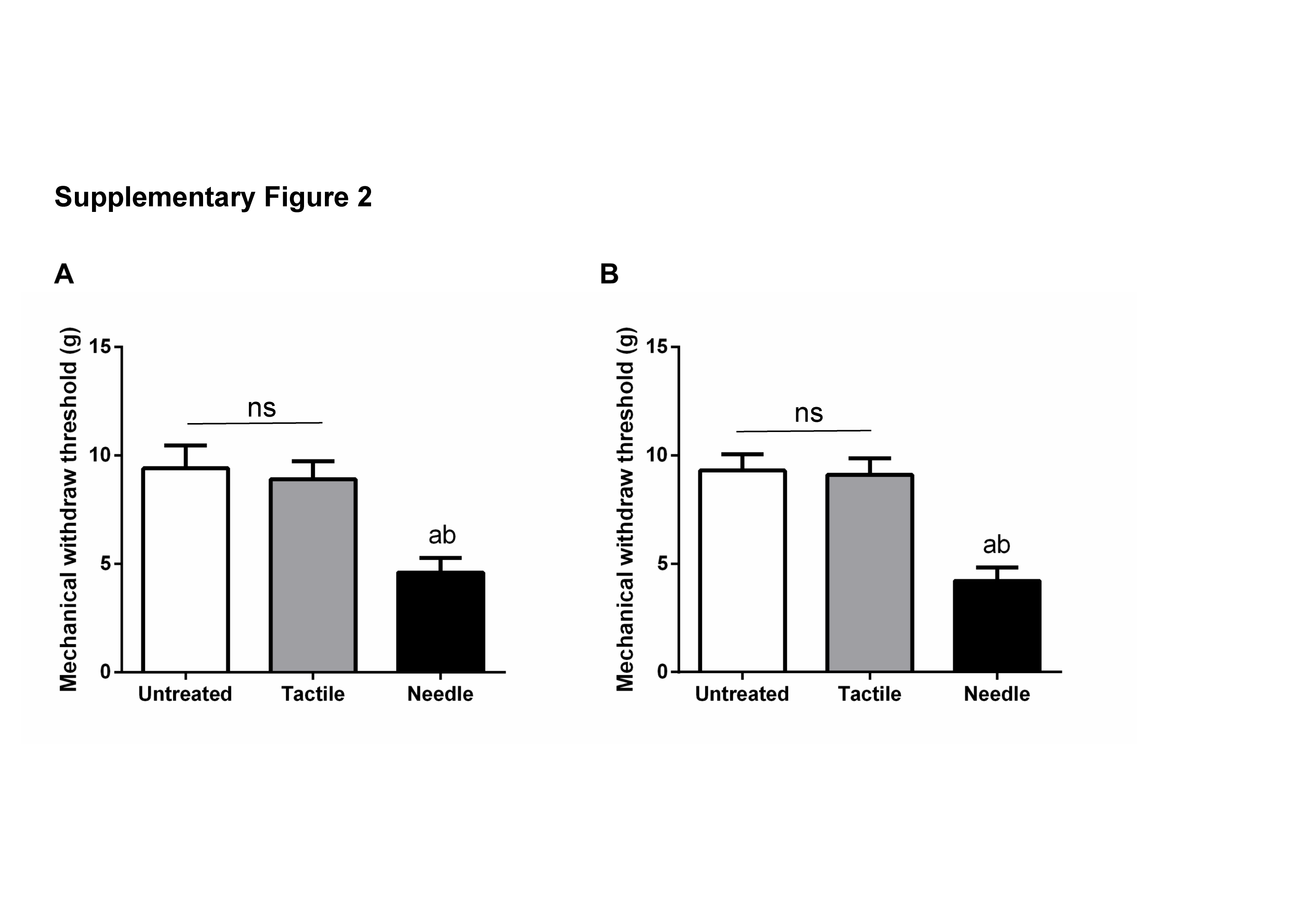

Supplement: Supplementary file 2 [file Image_2.tif]

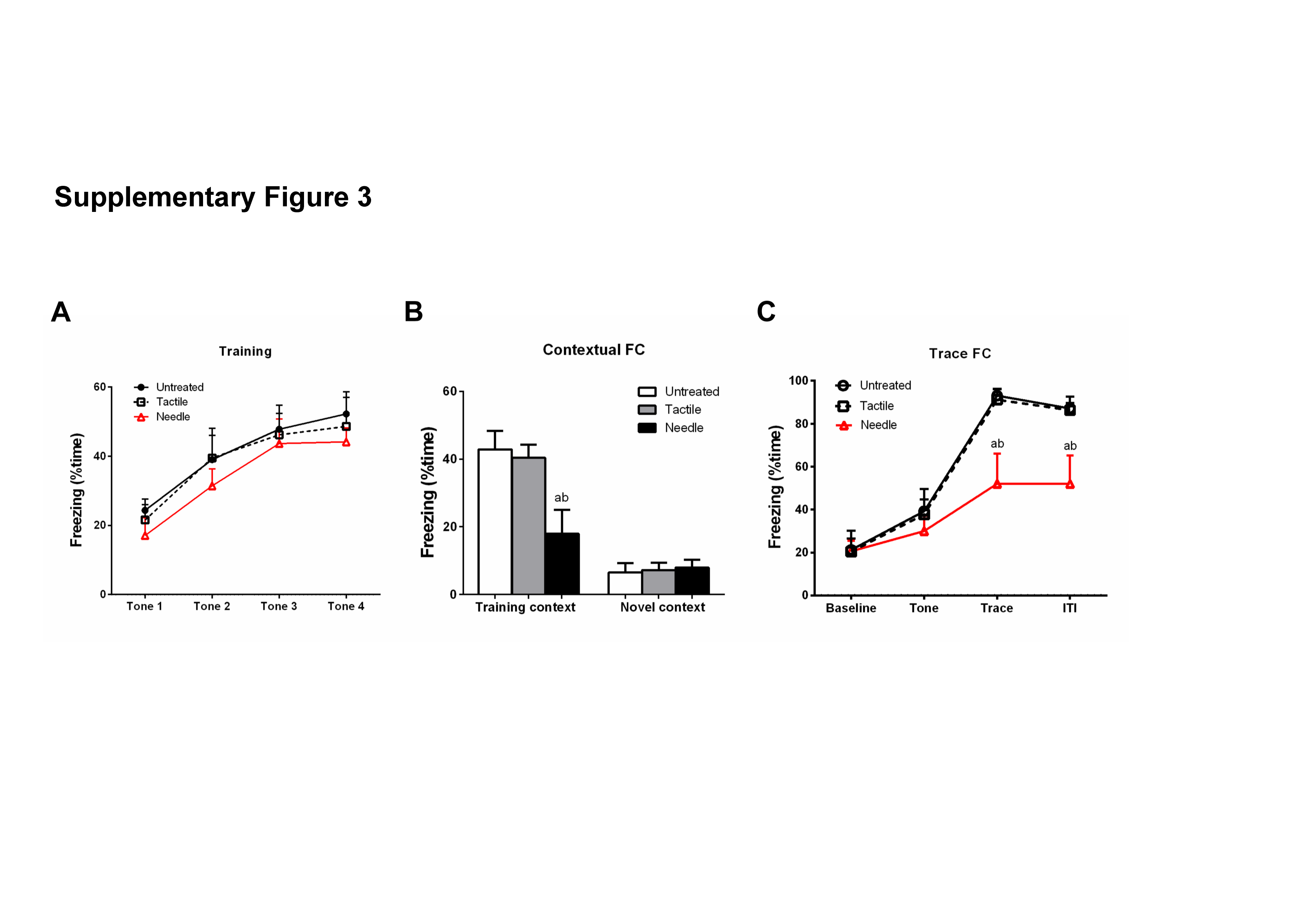

Supplement: Supplementary file 3 [file Image_3.tif]

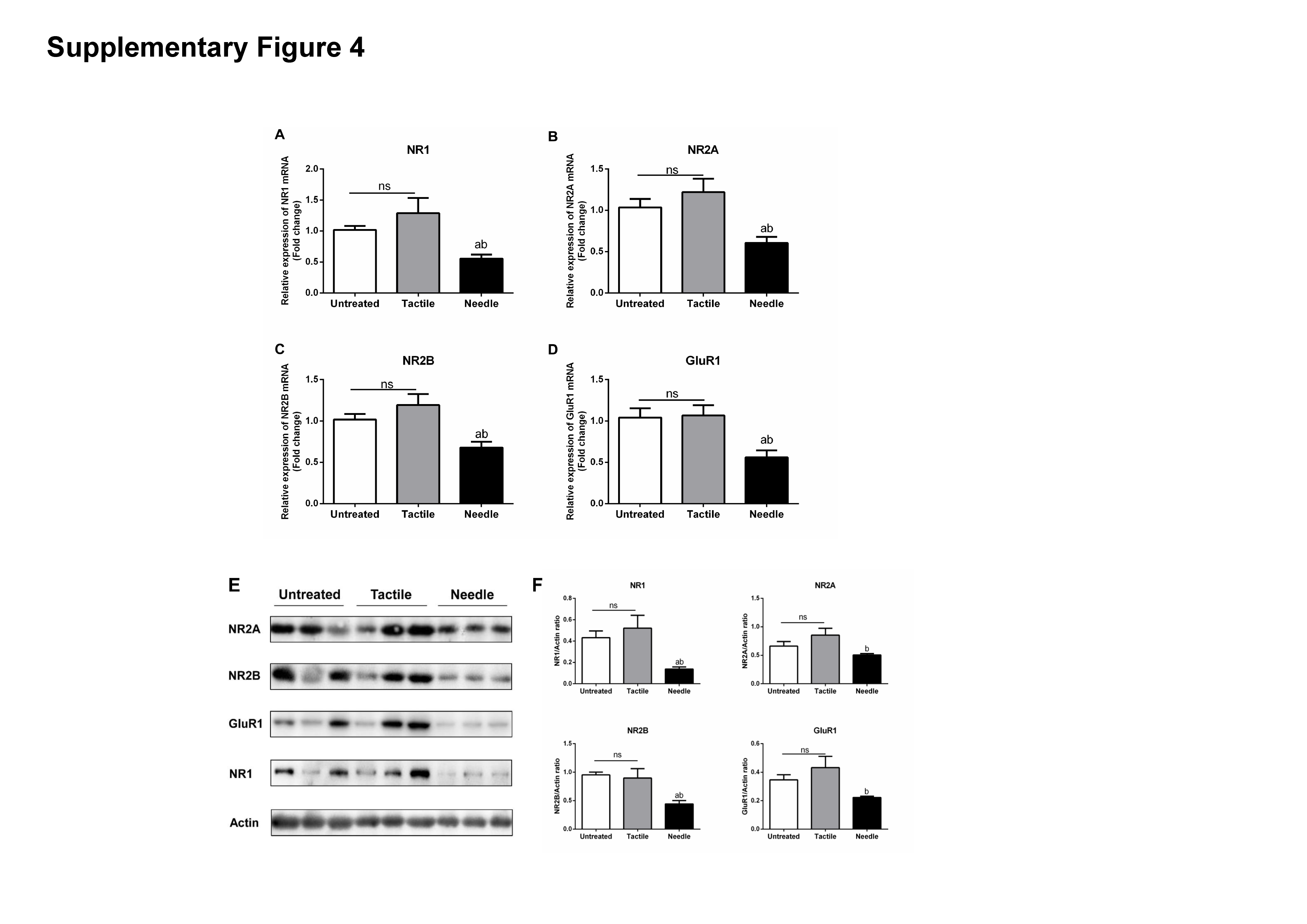

Supplement: Supplementary file 4 [file Image_4.tif]

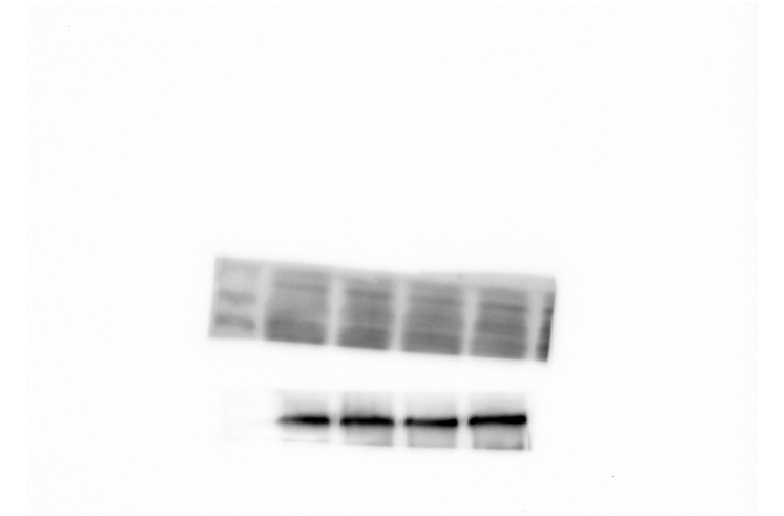

Supplement: Supplementary file 6 [file Data_Sheet_1.ZIP › raw data/WB/3w,act.jpg]

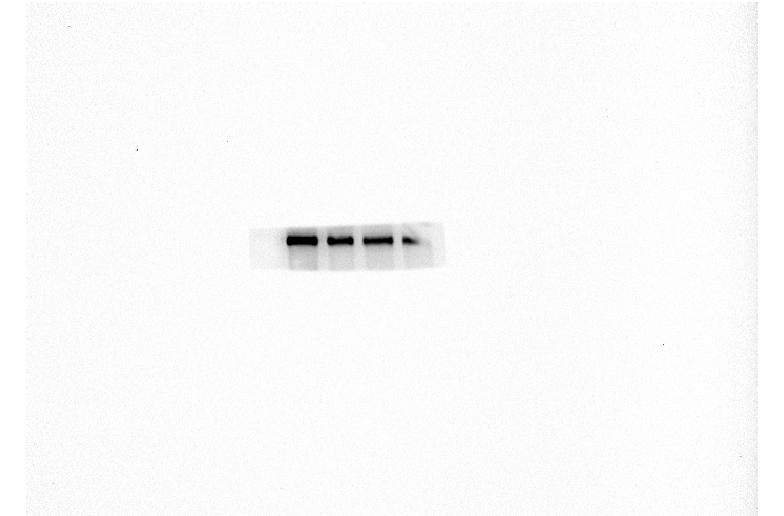

Supplement: Supplementary file 6 [file Data_Sheet_1.ZIP › raw data/WB/12w,glur1.jpg]

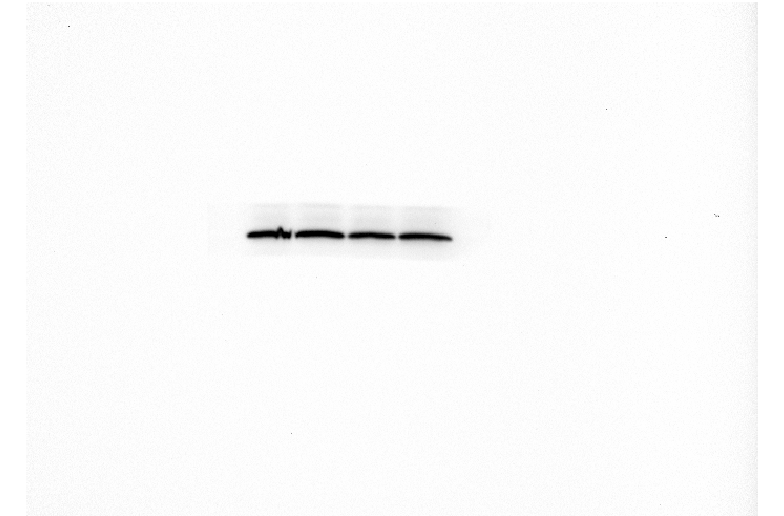

Supplement: Supplementary file 6 [file Data_Sheet_1.ZIP › raw data/WB/3w,actin.jpg]

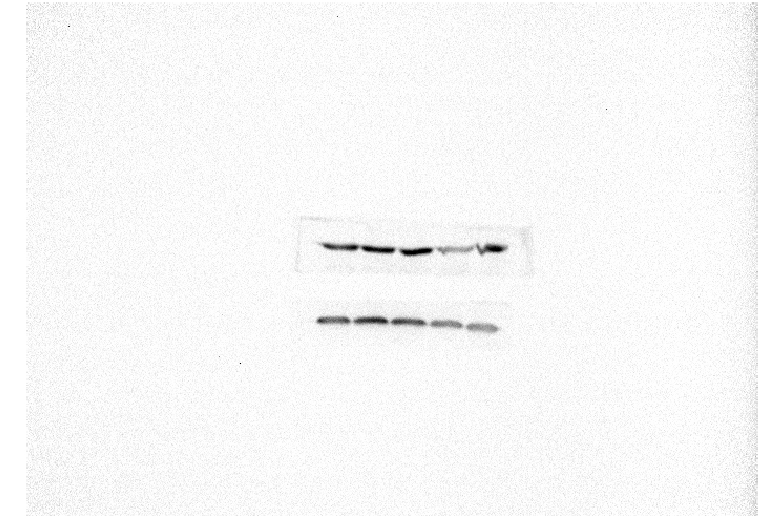

Supplement: Supplementary file 6 [file Data_Sheet_1.ZIP › raw data/WB/3w,a3.jpg]

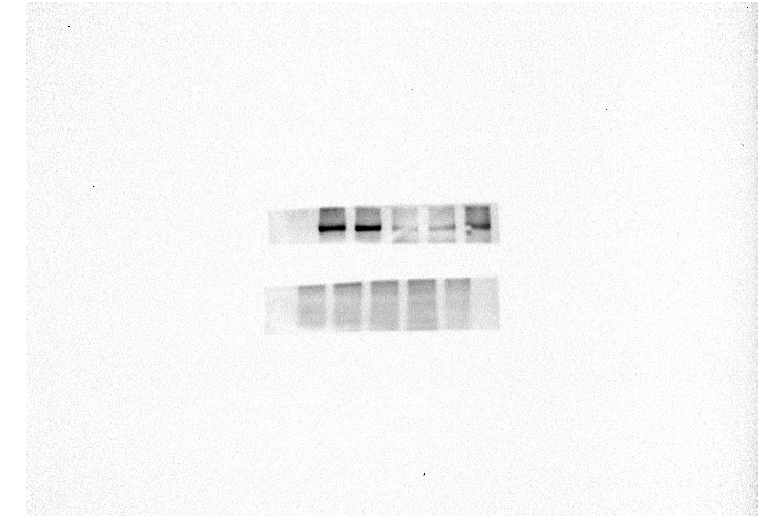

Supplement: Supplementary file 6 [file Data_Sheet_1.ZIP › raw data/WB/3w,nr2a.jpg]

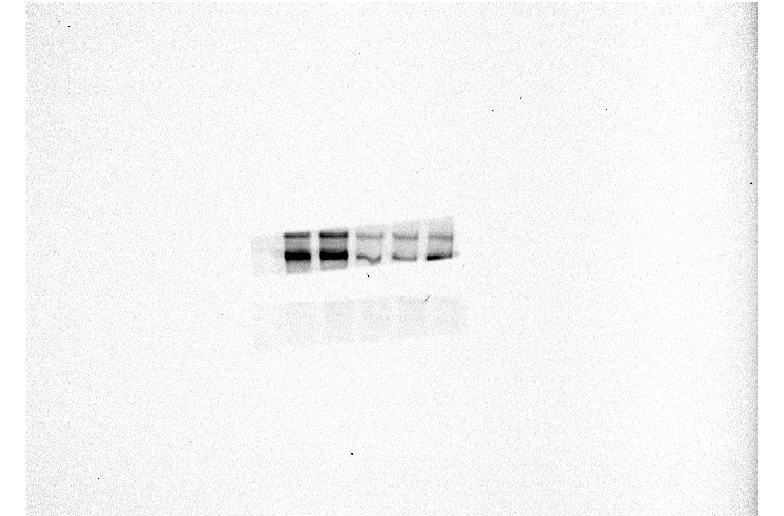

Supplement: Supplementary file 6 [file Data_Sheet_1.ZIP › raw data/WB/3w,nr2b.jpg]

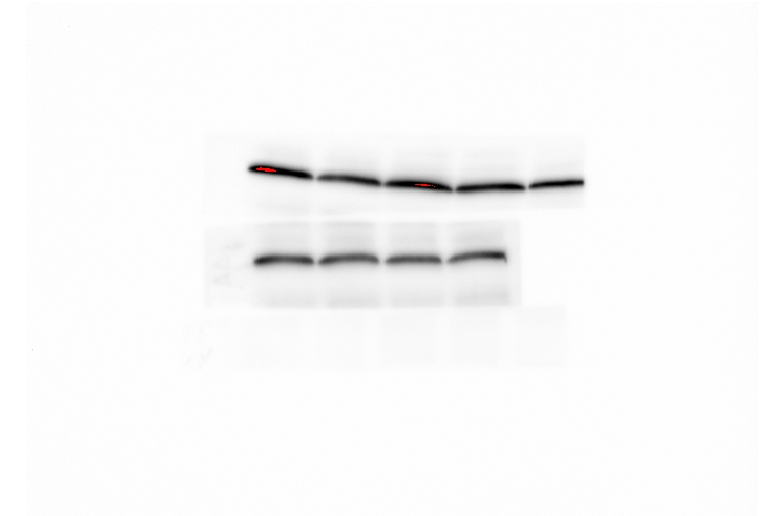

Supplement: Supplementary file 6 [file Data_Sheet_1.ZIP › raw data/WB/3w,glur1.jpg]

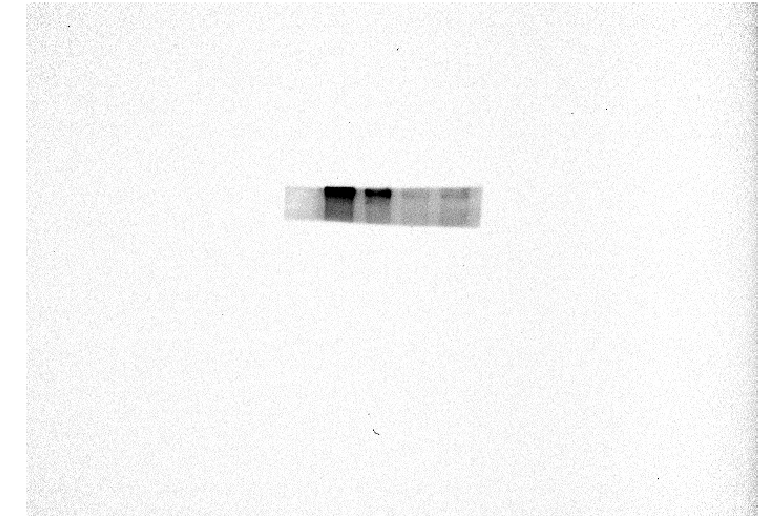

Supplement: Supplementary file 6 [file Data_Sheet_1.ZIP › raw data/WB/12w,nr2b.jpg]

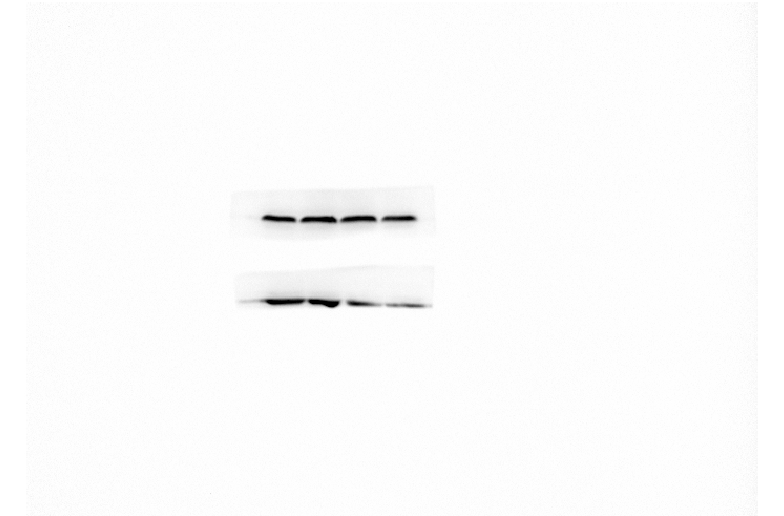

Supplement: Supplementary file 6 [file Data_Sheet_1.ZIP › raw data/WB/112w,actin.jpg]

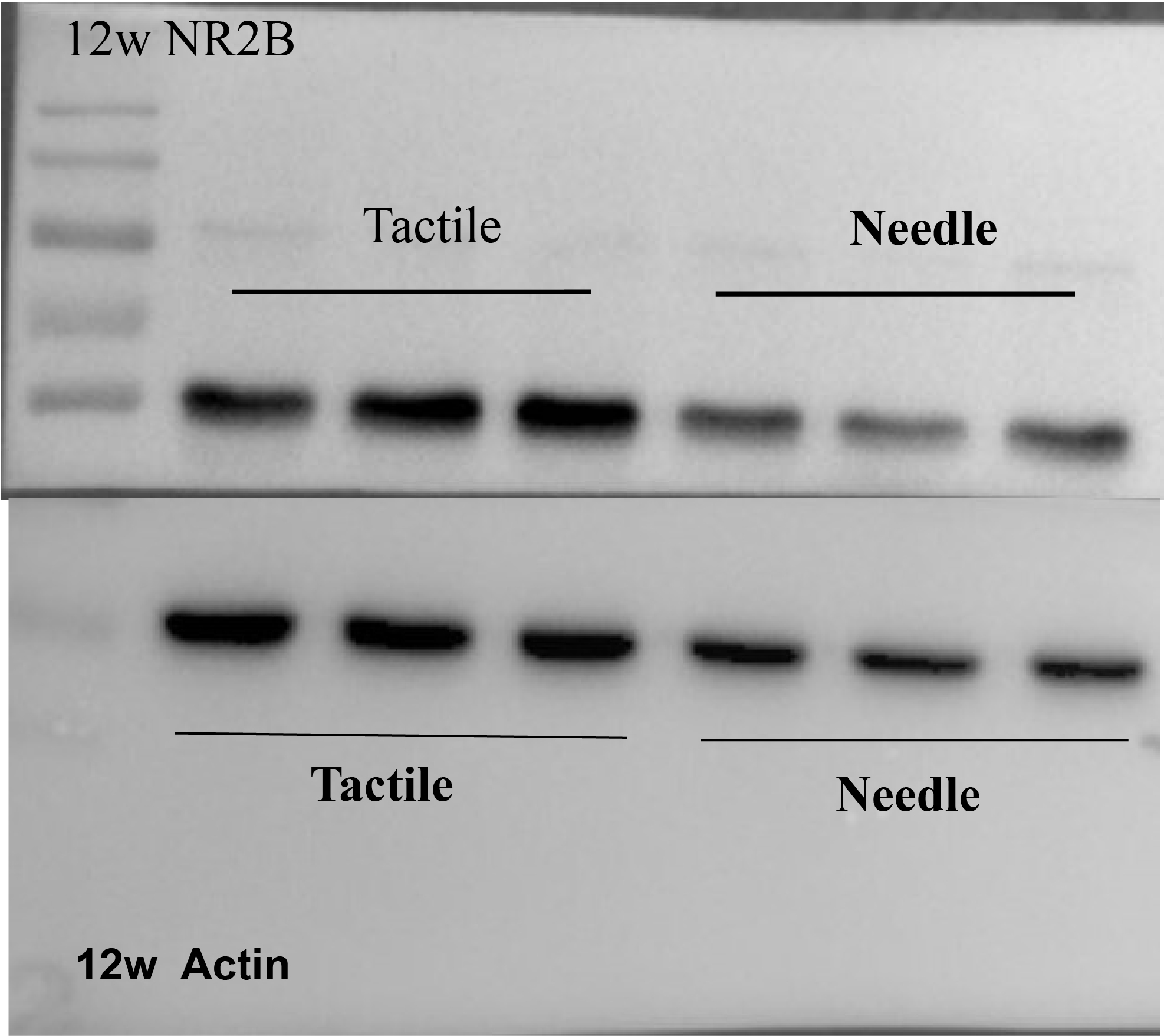

Supplement: Supplementary file 7 [file Data_Sheet_2.ZIP › 12W/12w actin+NR2B.jpg]

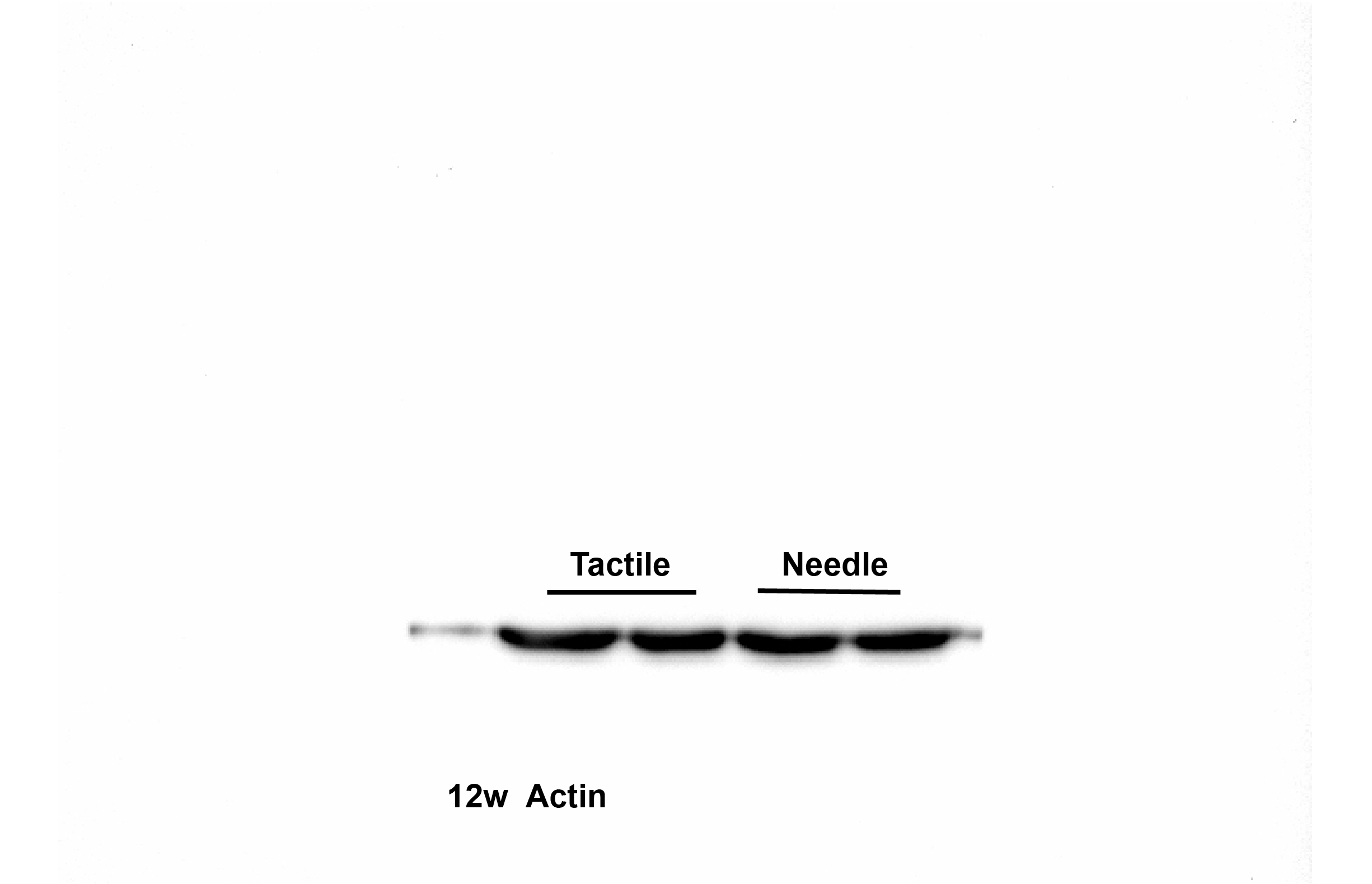

Supplement: Supplementary file 7 [file Data_Sheet_2.ZIP › 12W/12w,Actin-1.jpg]

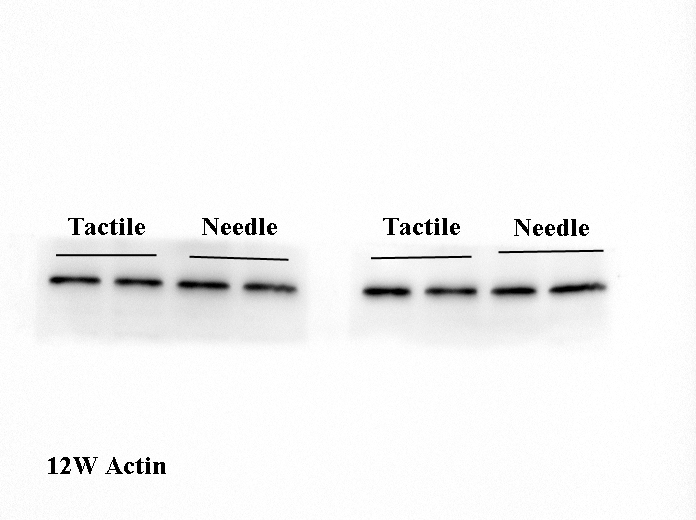

Supplement: Supplementary file 7 [file Data_Sheet_2.ZIP › 12W/12w,Actin-2.jpg]

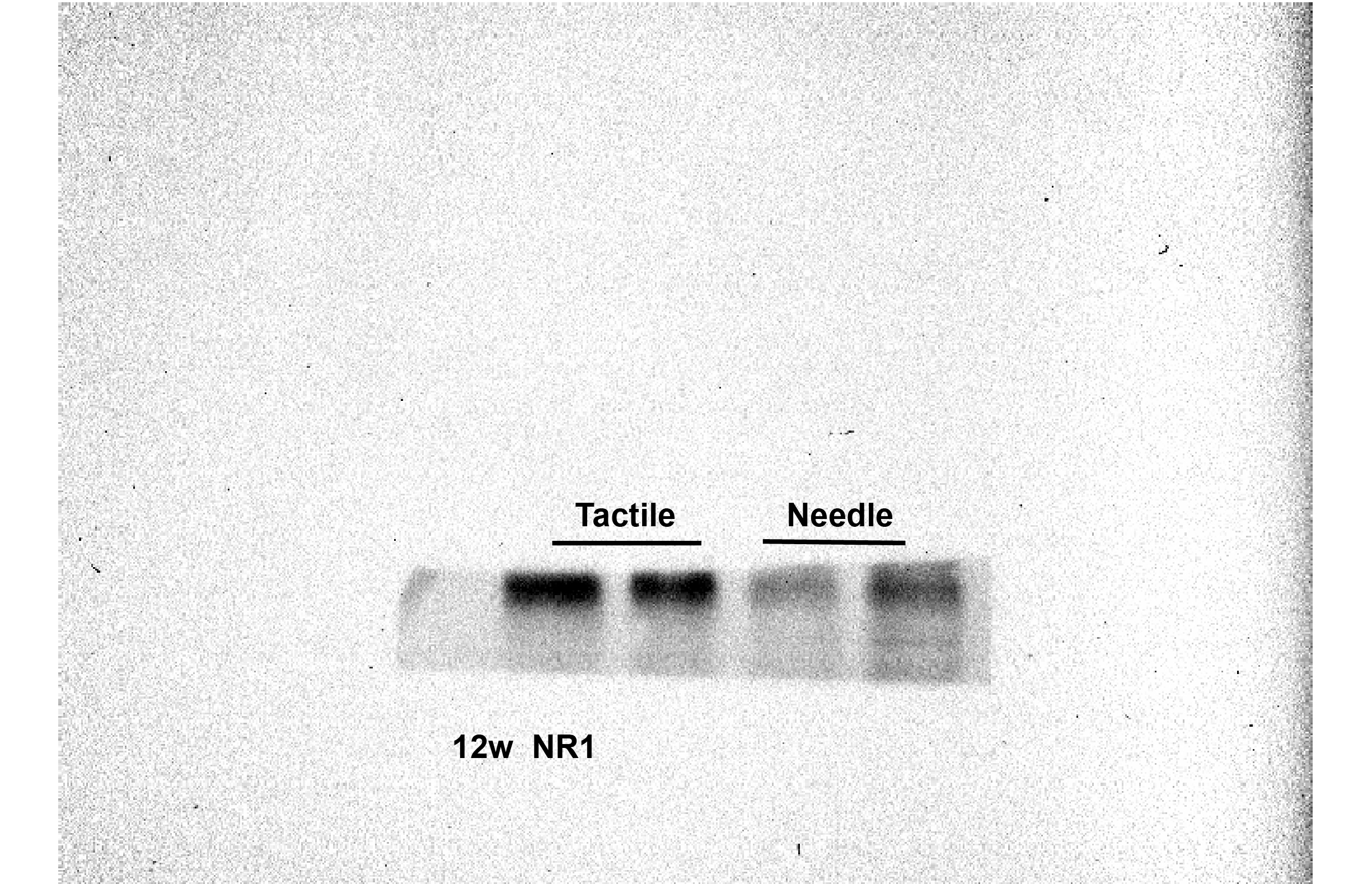

Supplement: Supplementary file 7 [file Data_Sheet_2.ZIP › 12W/12w,NR1-1.jpg]

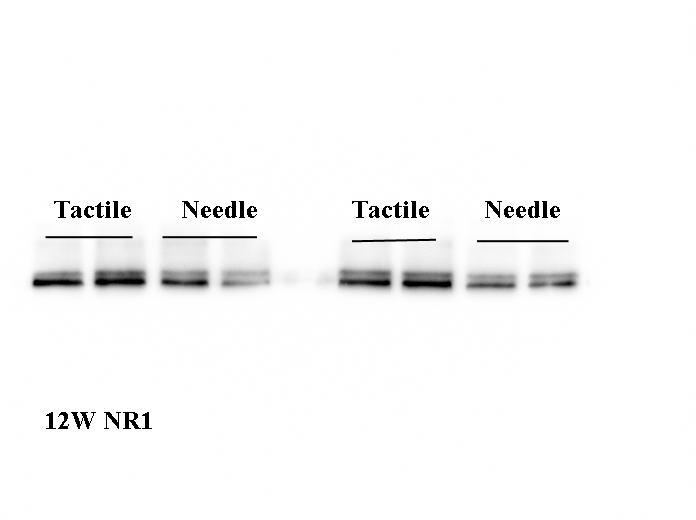

Supplement: Supplementary file 7 [file Data_Sheet_2.ZIP › 12W/12w,NR1-2.jpg]

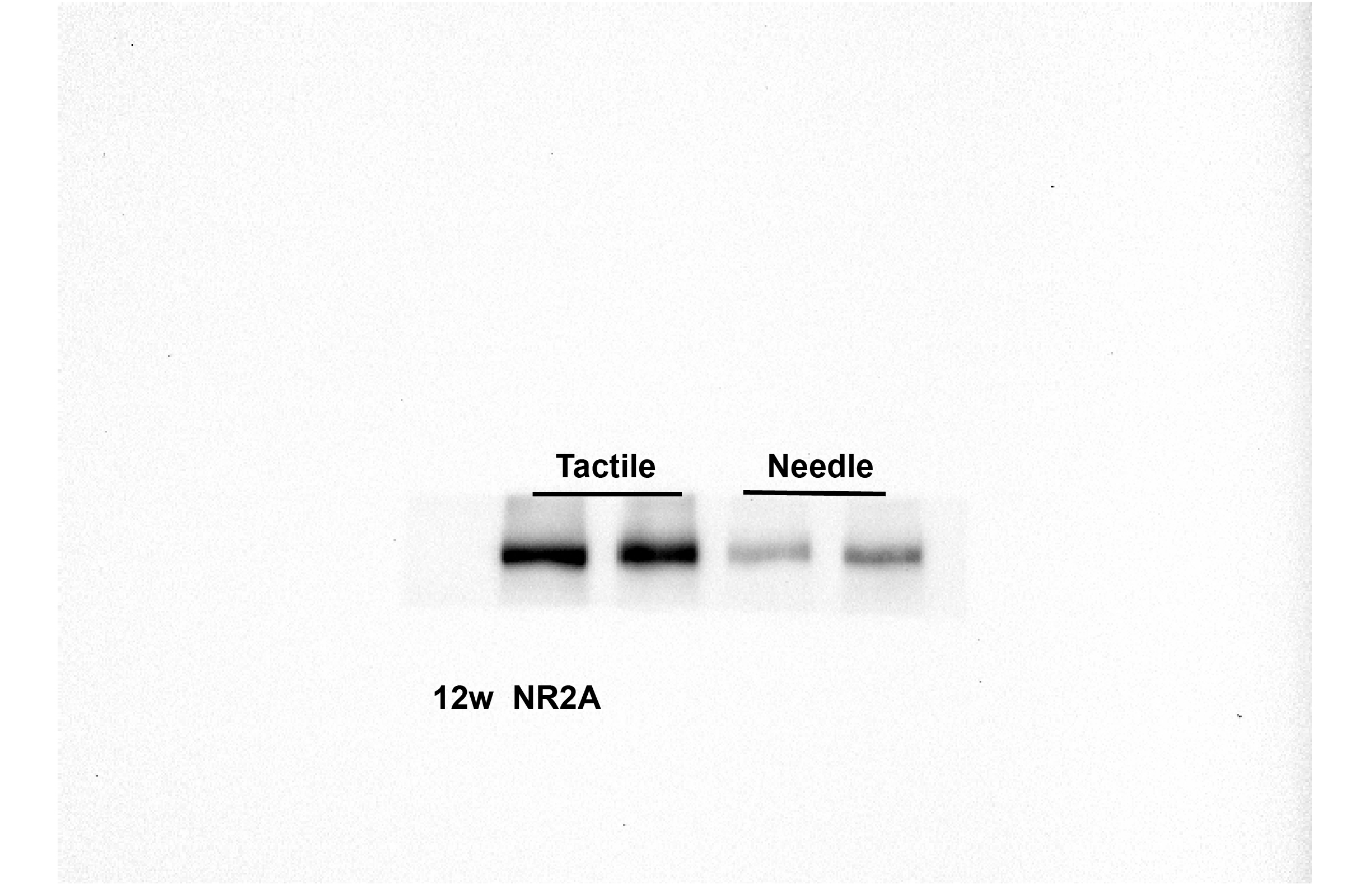

Supplement: Supplementary file 7 [file Data_Sheet_2.ZIP › 12W/12w,NR2A-1.jpg]

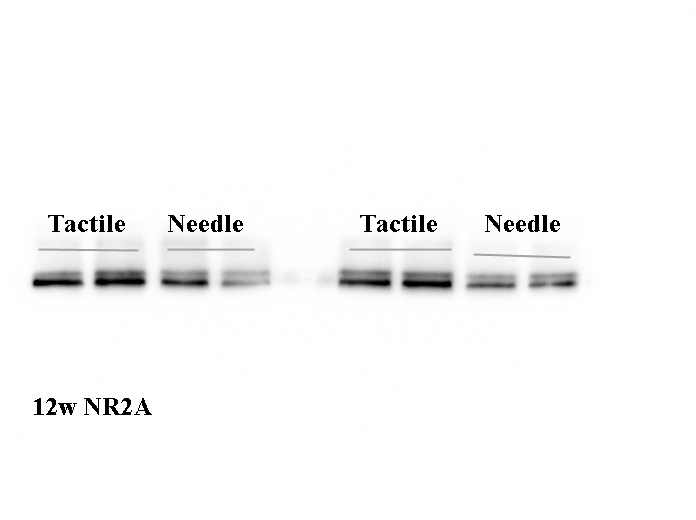

Supplement: Supplementary file 7 [file Data_Sheet_2.ZIP › 12W/12w,NR2A-2.jpg]

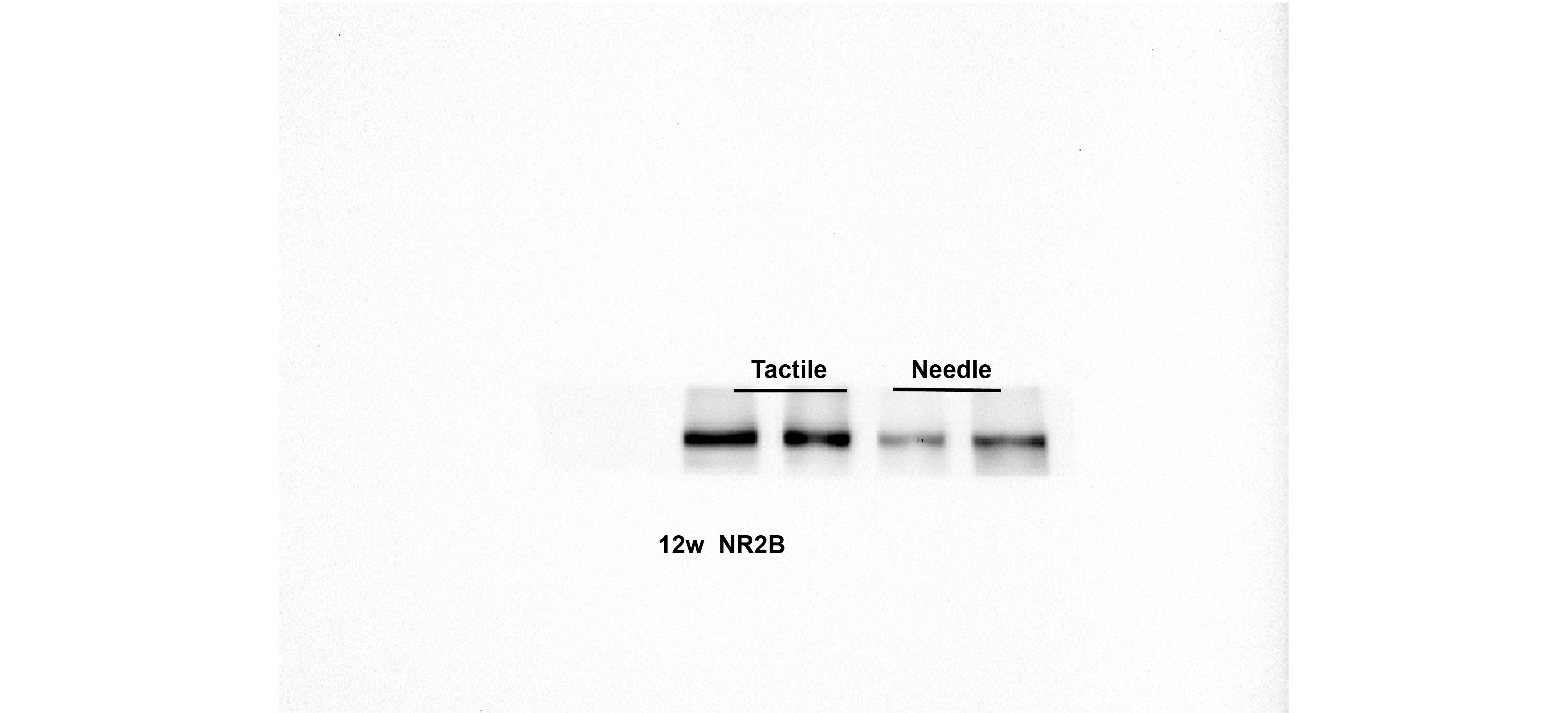

Supplement: Supplementary file 7 [file Data_Sheet_2.ZIP › 12W/12w,NR2B-1.jpg]

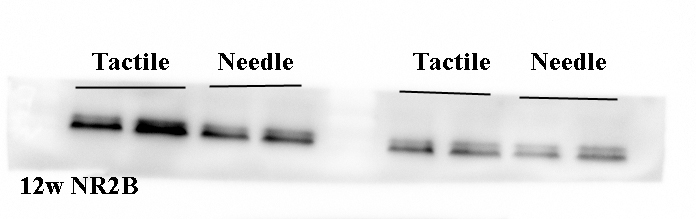

Supplement: Supplementary file 7 [file Data_Sheet_2.ZIP › 12W/12w,NR2B-2.jpg]

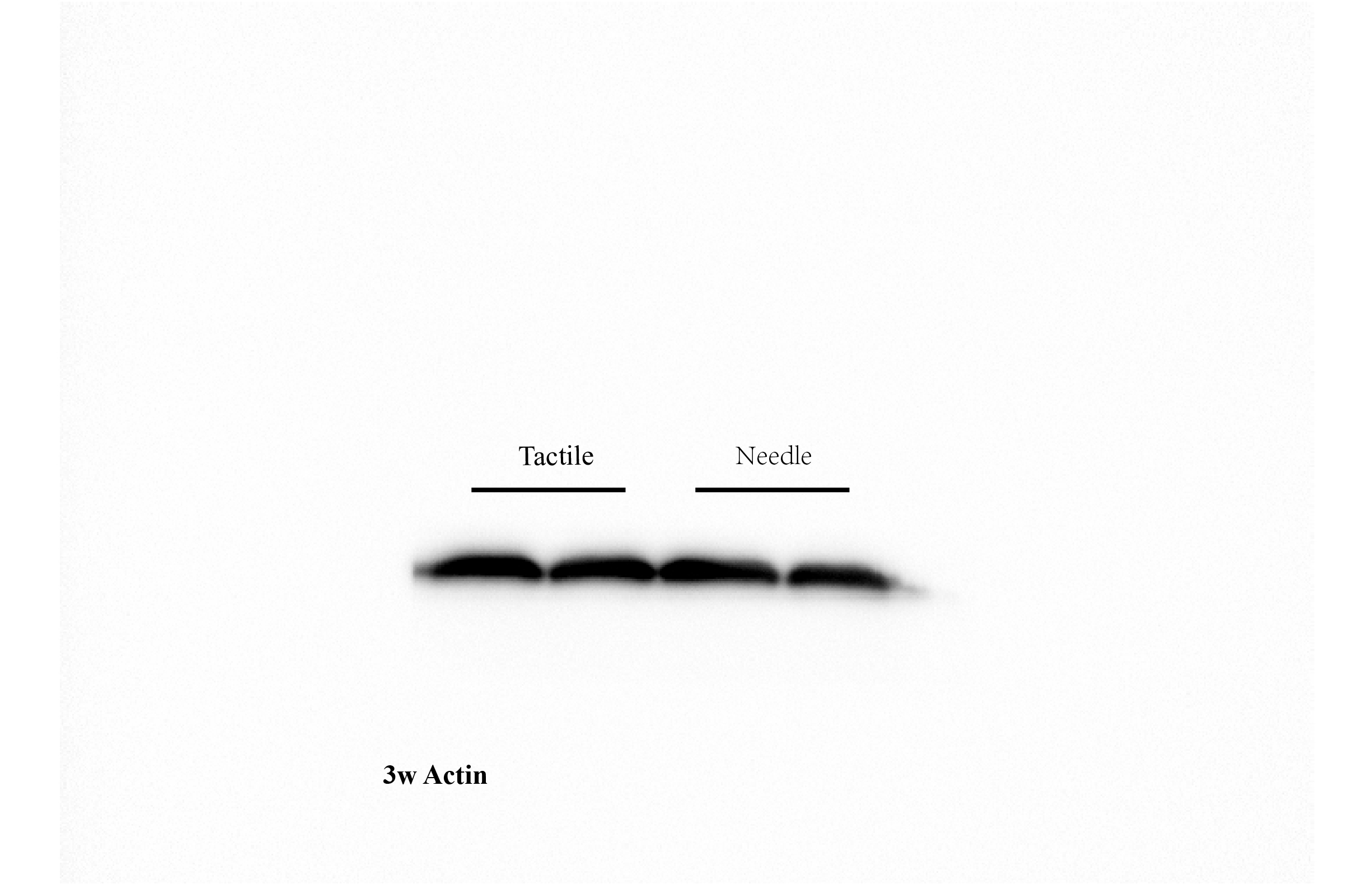

Supplement: Supplementary file 7 [file Data_Sheet_2.ZIP › 3W/3W, Actin-1.jpg]

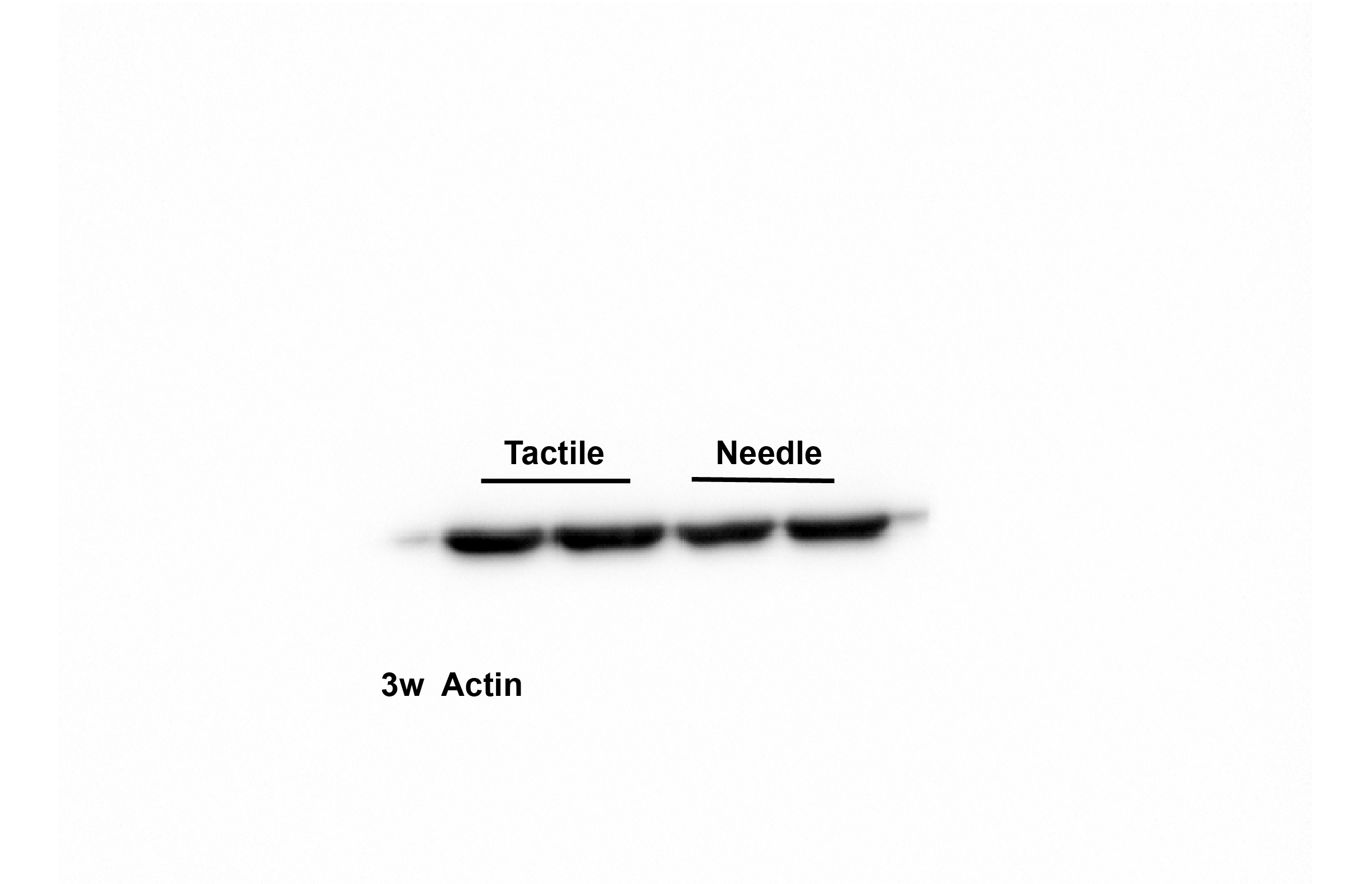

Supplement: Supplementary file 7 [file Data_Sheet_2.ZIP › 3W/3W, Actin-2.jpg]

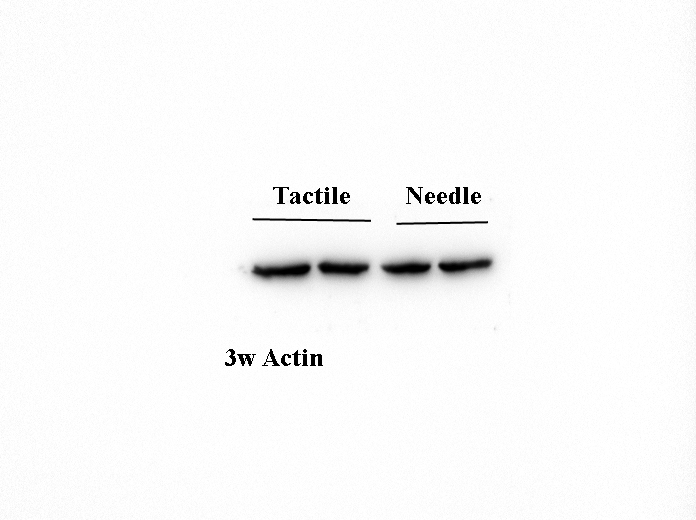

Supplement: Supplementary file 7 [file Data_Sheet_2.ZIP › 3W/3W, Actin-3.jpg]

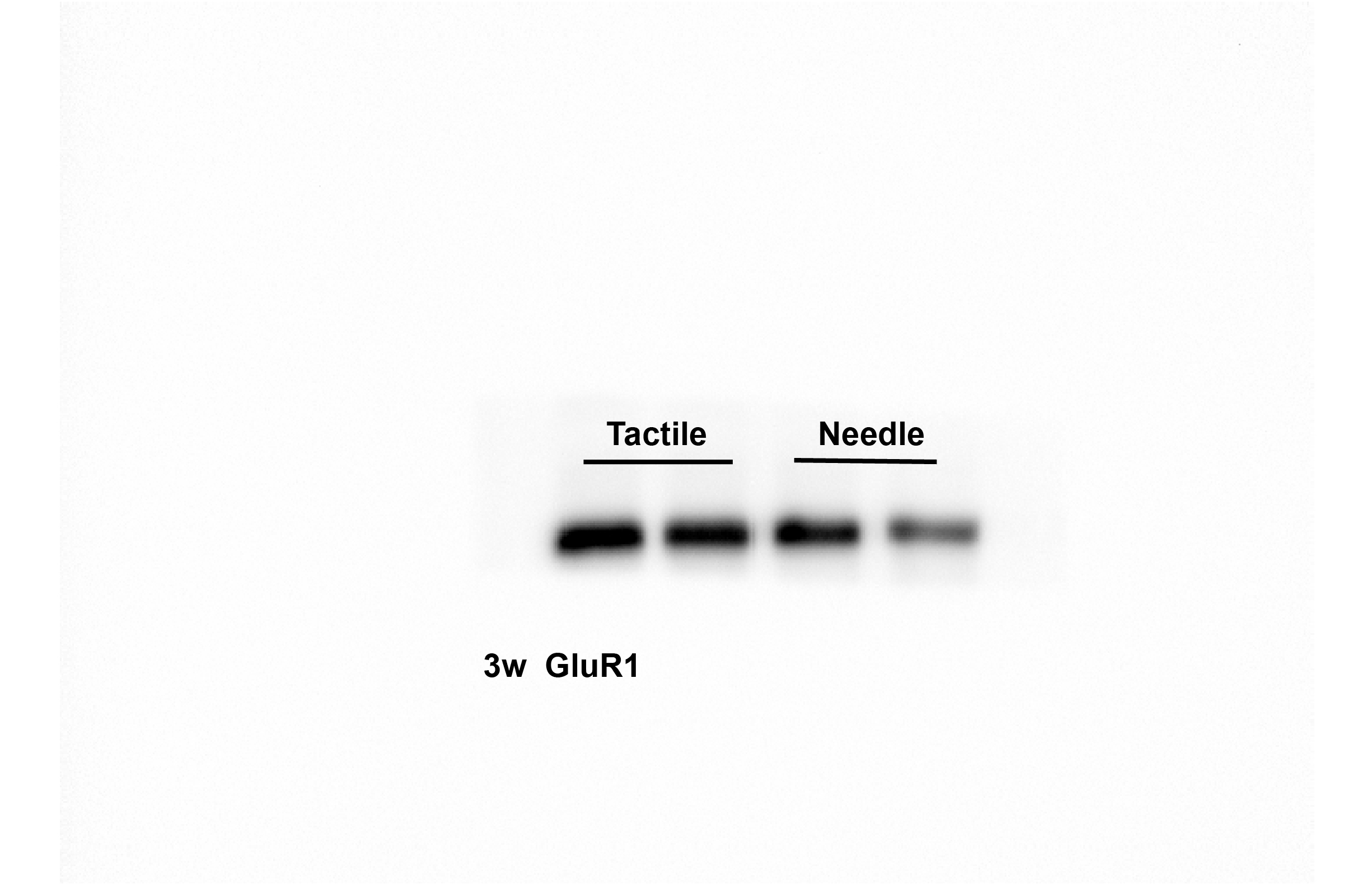

Supplement: Supplementary file 7 [file Data_Sheet_2.ZIP › 3W/3W,GluR1-1.jpg]

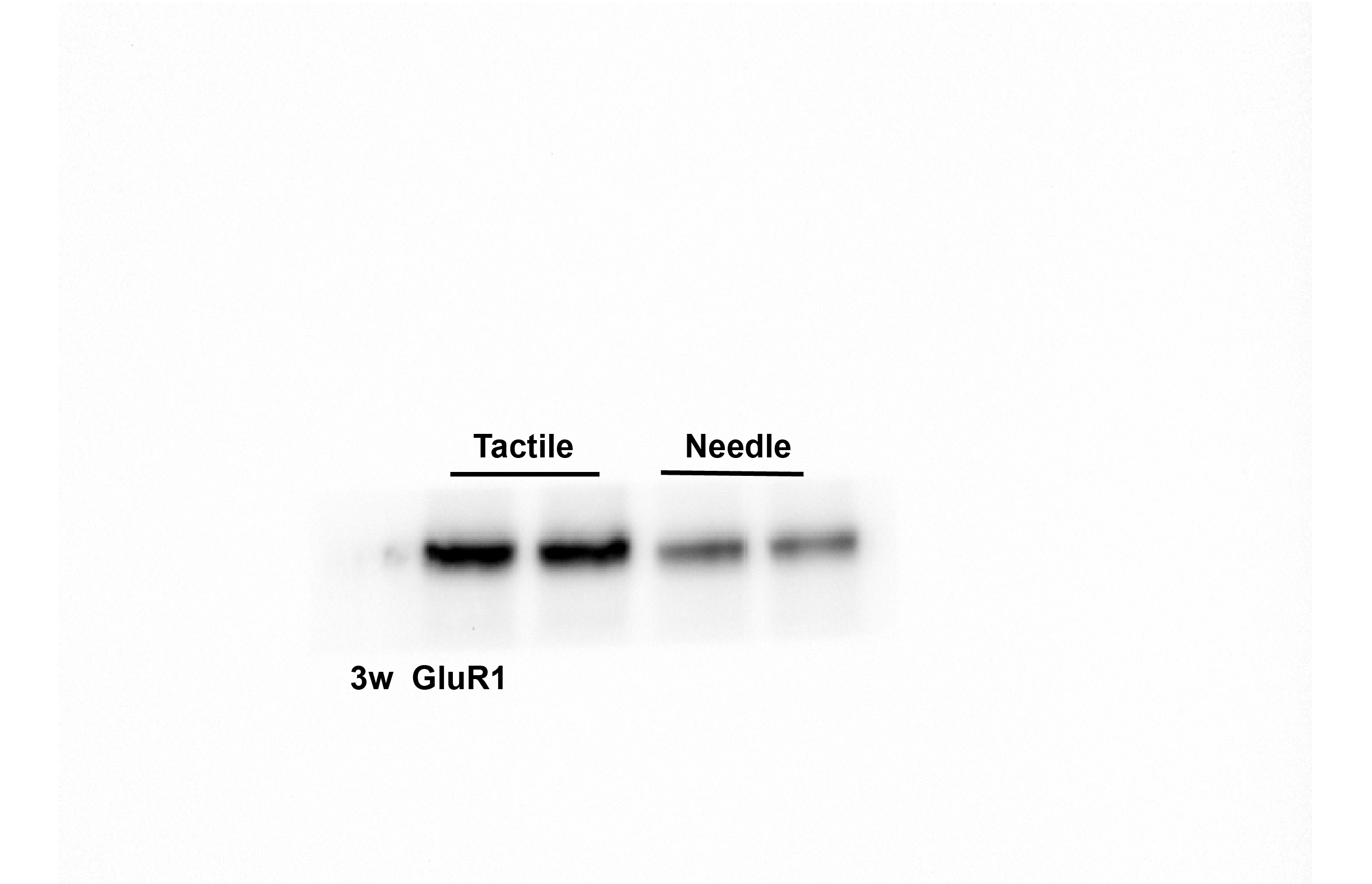

Supplement: Supplementary file 7 [file Data_Sheet_2.ZIP › 3W/3W,GluR1-2.jpg]

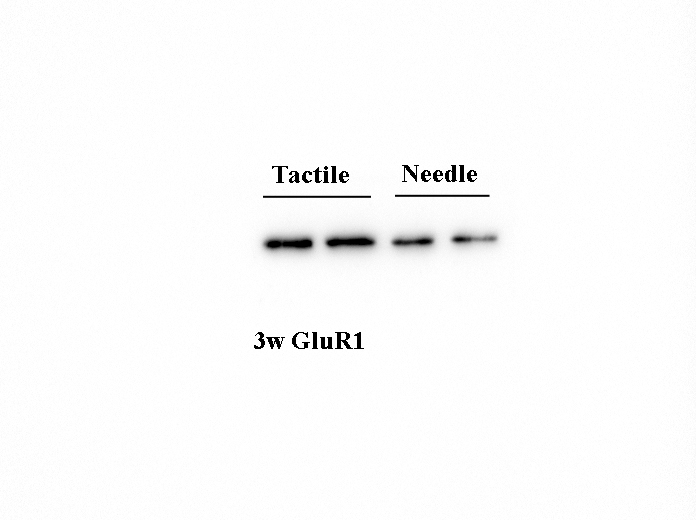

Supplement: Supplementary file 7 [file Data_Sheet_2.ZIP › 3W/3W,GluR1-3.jpg]

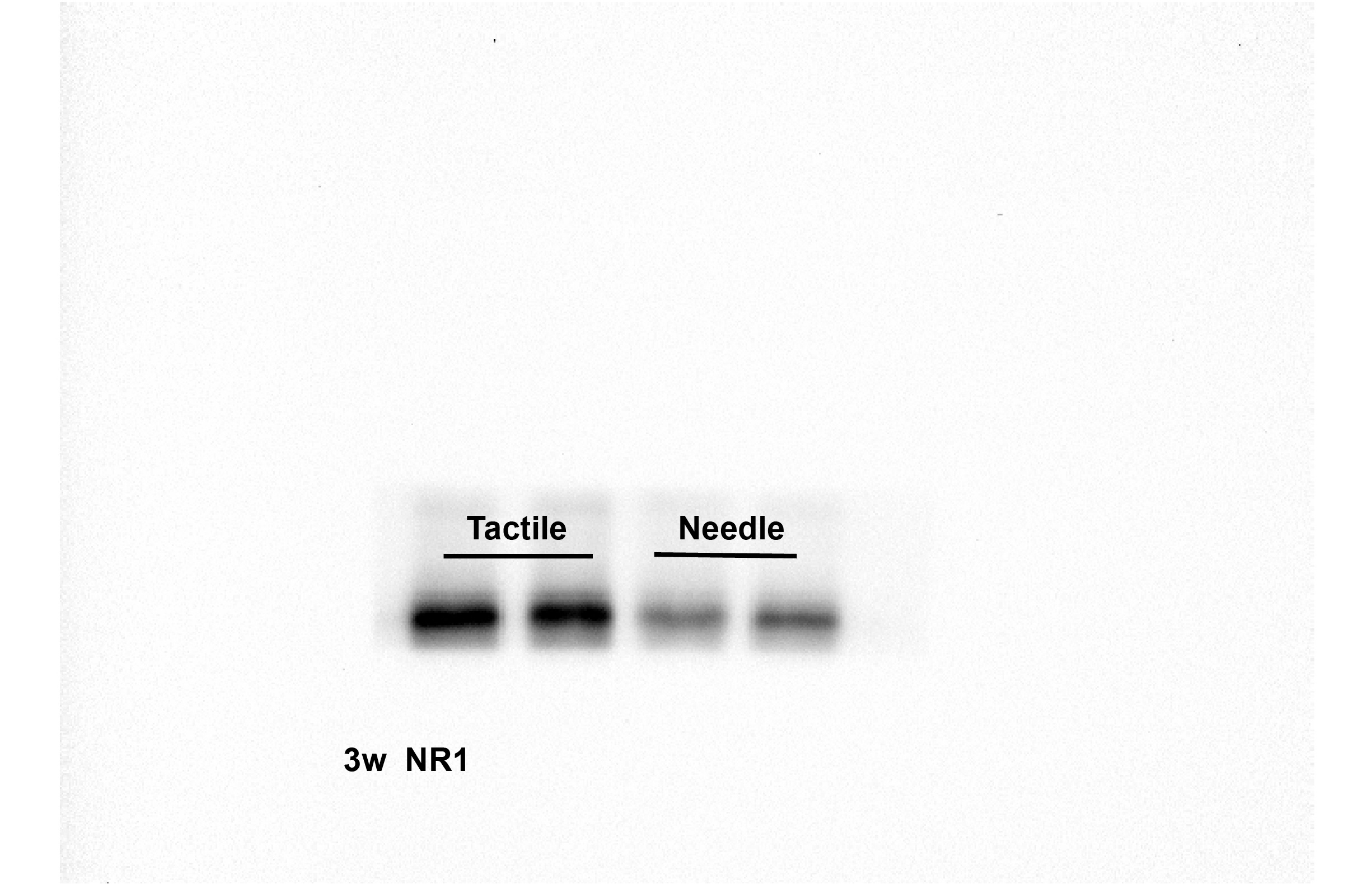

Supplement: Supplementary file 7 [file Data_Sheet_2.ZIP › 3W/3W,NR1-1.jpg]

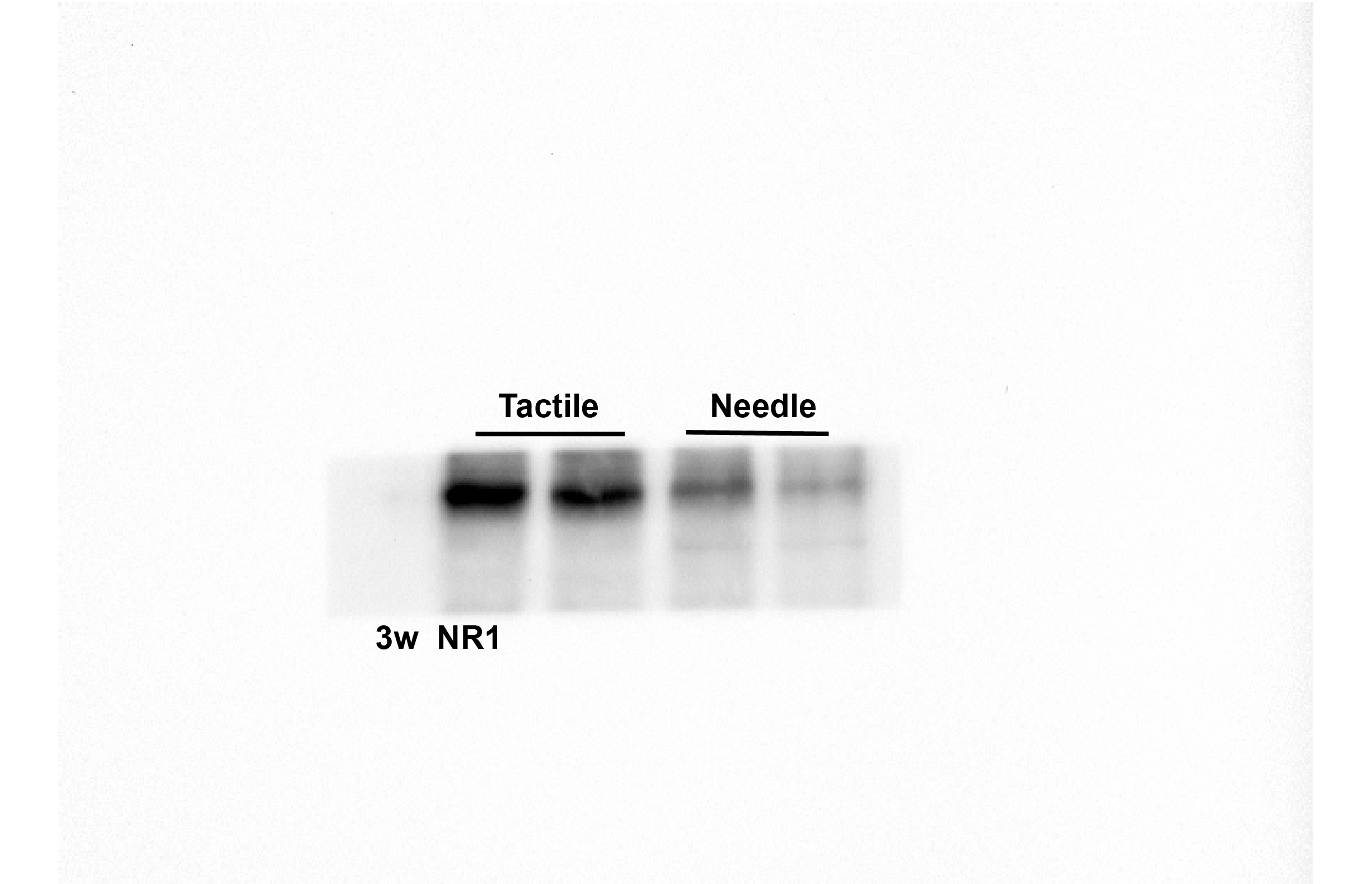

Supplement: Supplementary file 7 [file Data_Sheet_2.ZIP › 3W/3W,NR1-2.jpg]

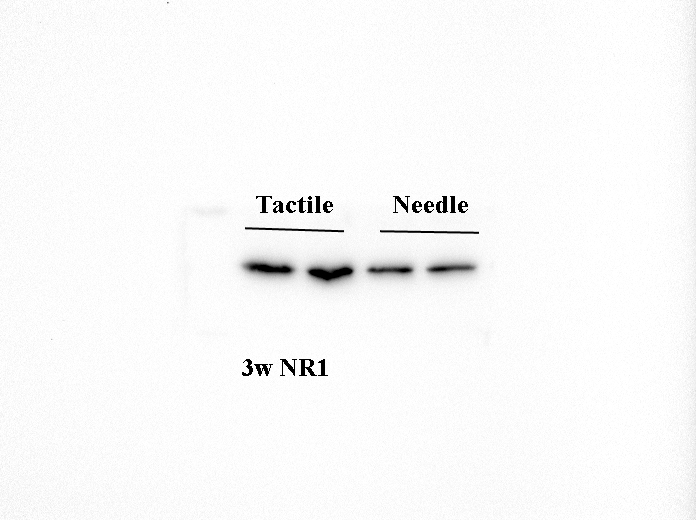

Supplement: Supplementary file 7 [file Data_Sheet_2.ZIP › 3W/3W,NR1-3.jpg]

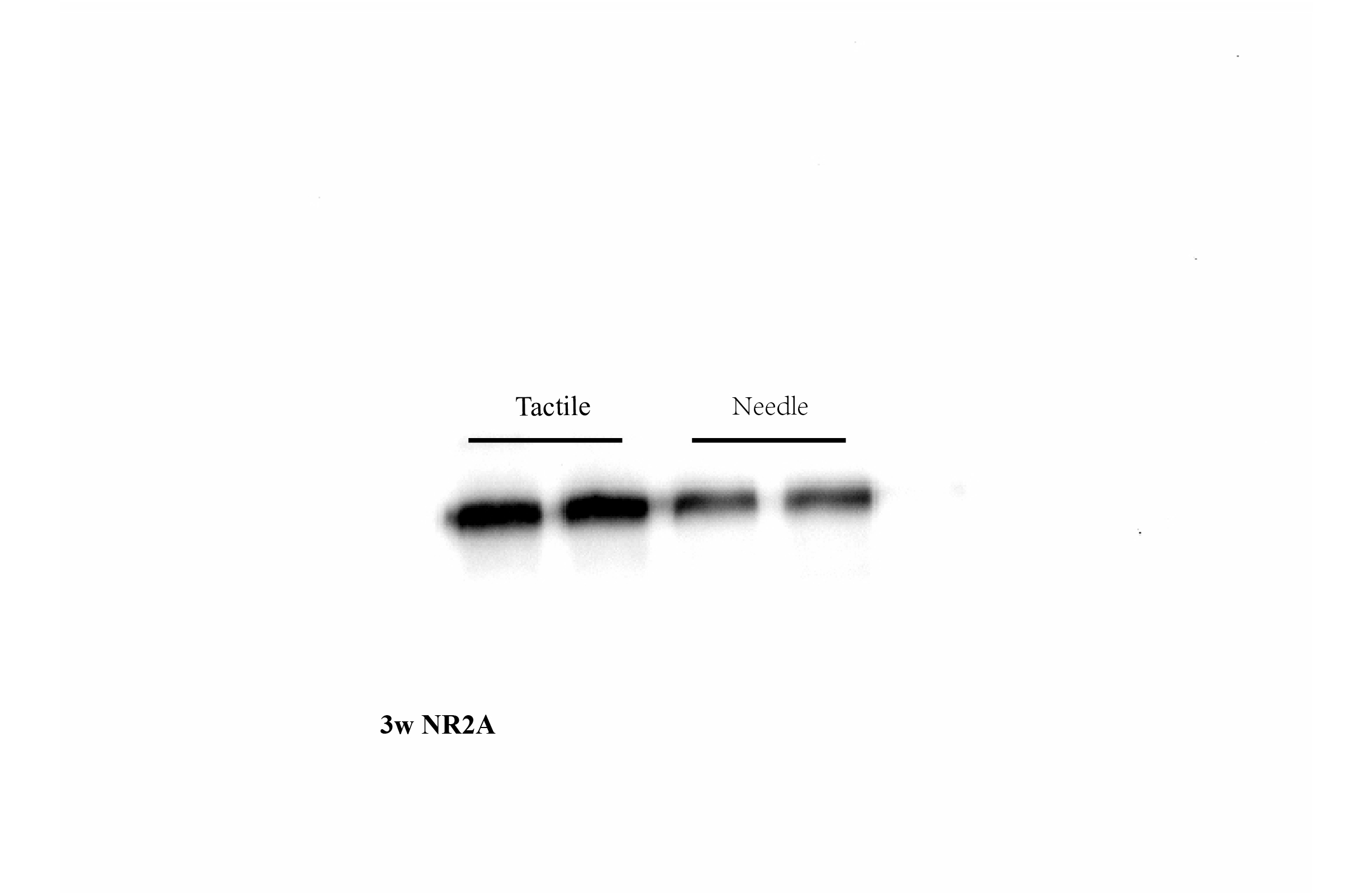

Supplement: Supplementary file 7 [file Data_Sheet_2.ZIP › 3W/3W,NR2A-1.jpg]

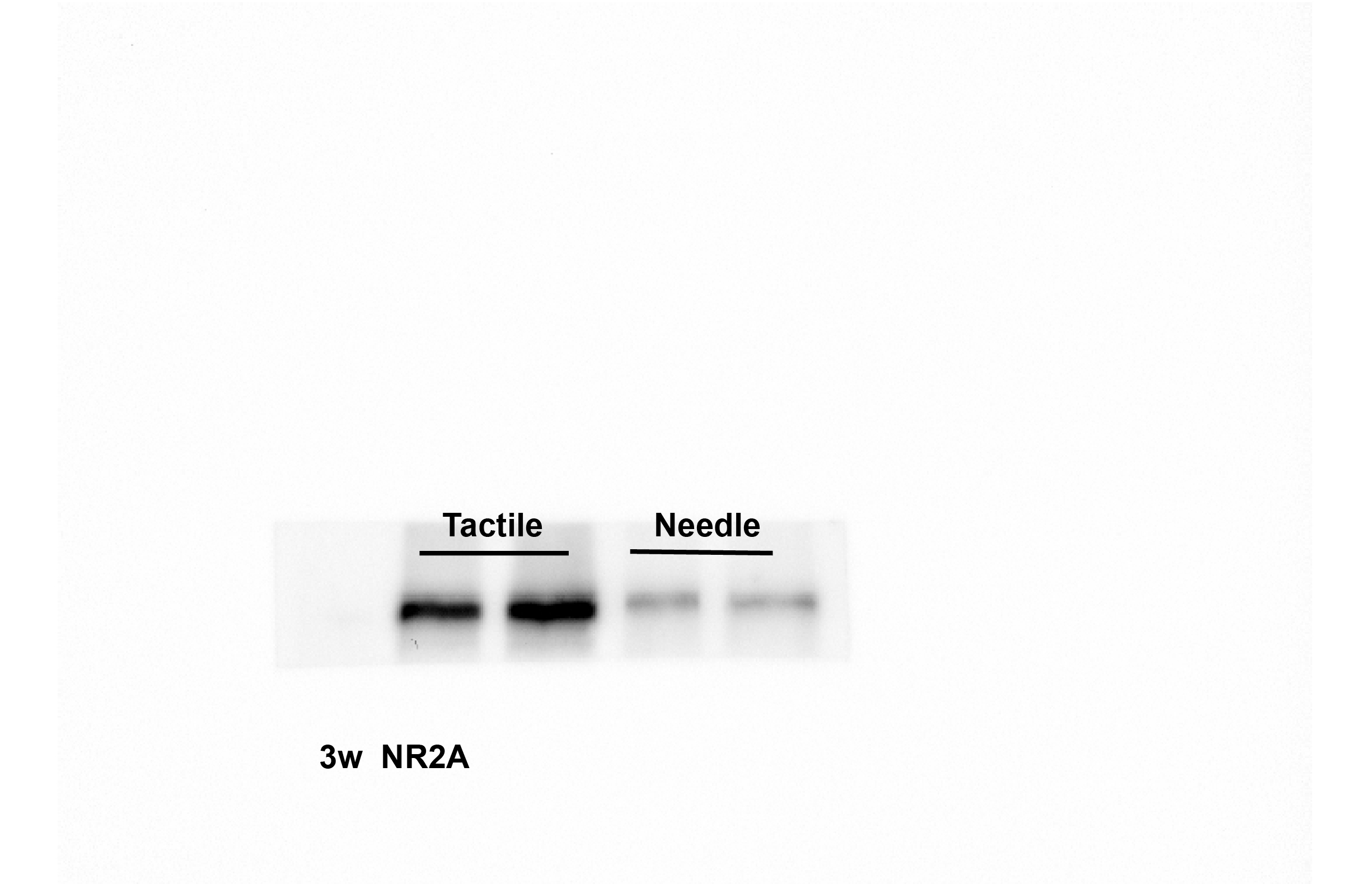

Supplement: Supplementary file 7 [file Data_Sheet_2.ZIP › 3W/3W,NR2A-2.jpg]

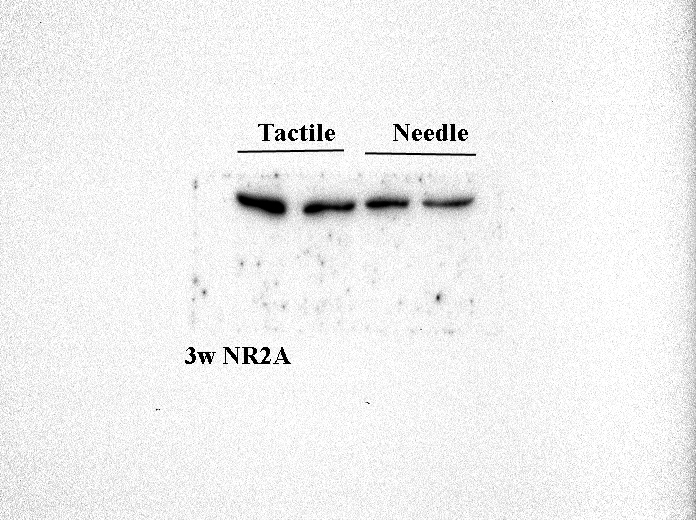

Supplement: Supplementary file 7 [file Data_Sheet_2.ZIP › 3W/3W,NR2A-3.jpg]

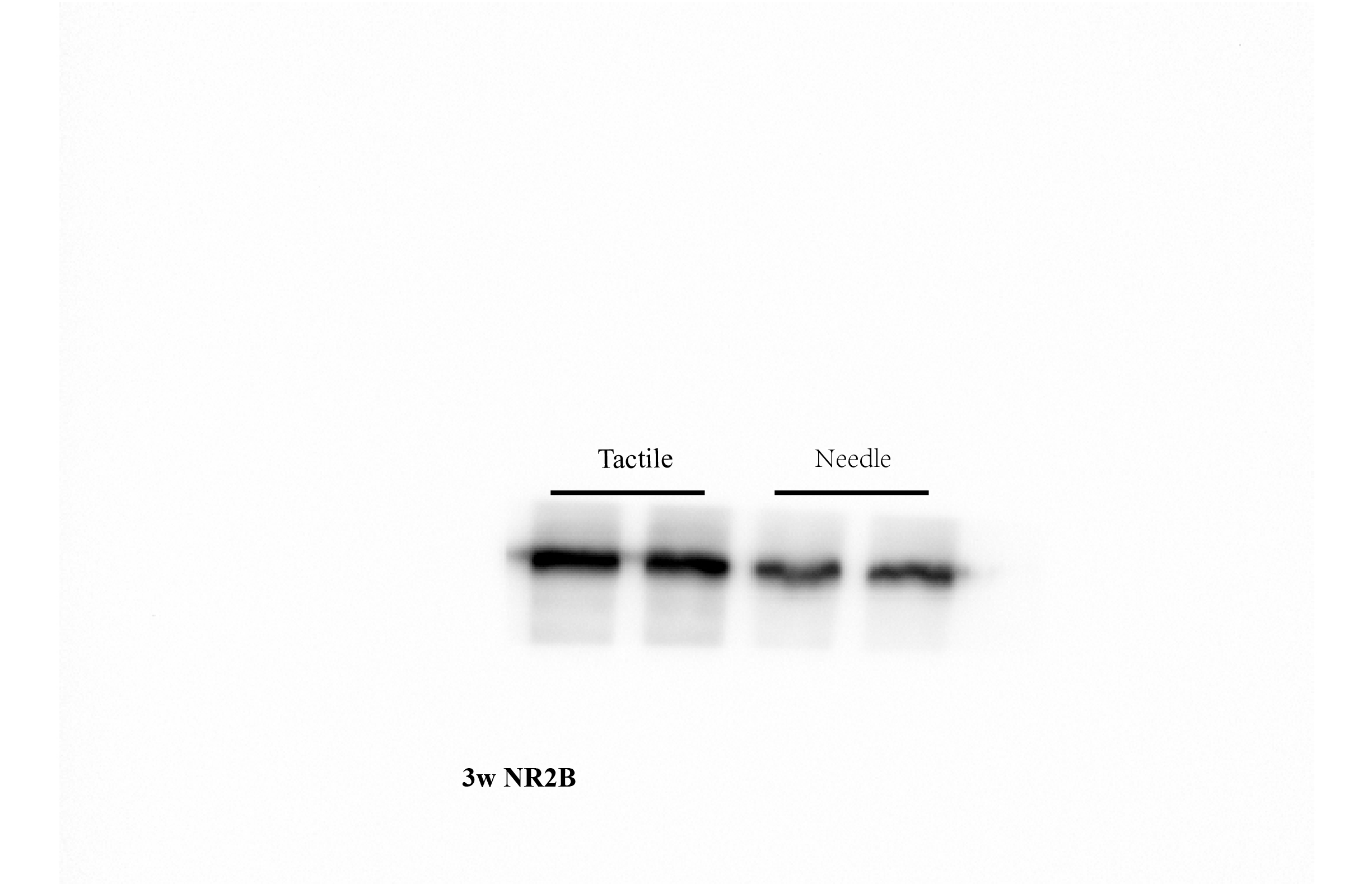

Supplement: Supplementary file 7 [file Data_Sheet_2.ZIP › 3W/3W,NR2B-1.jpg]

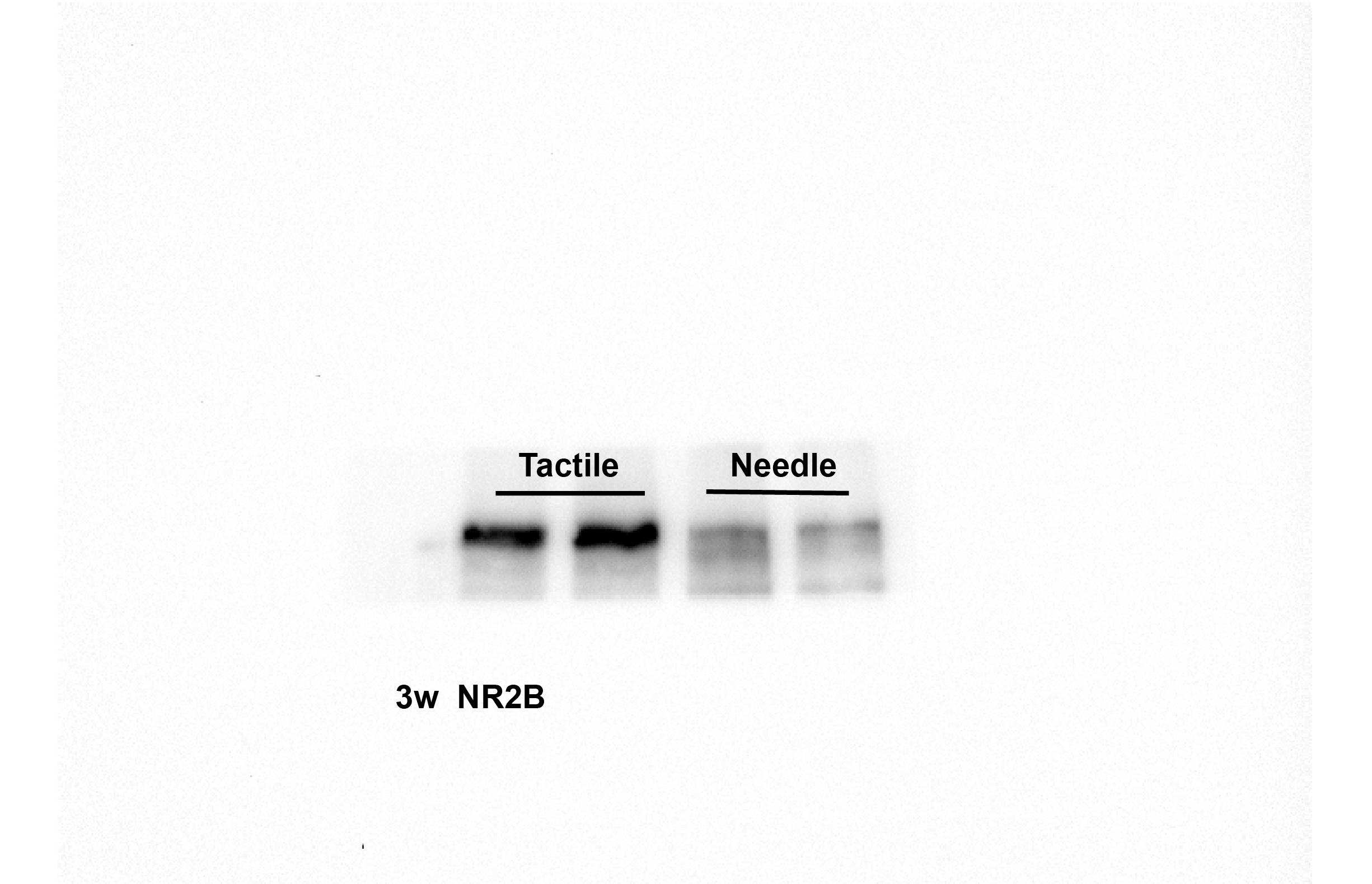

Supplement: Supplementary file 7 [file Data_Sheet_2.ZIP › 3W/3W,NR2B-2.jpg]

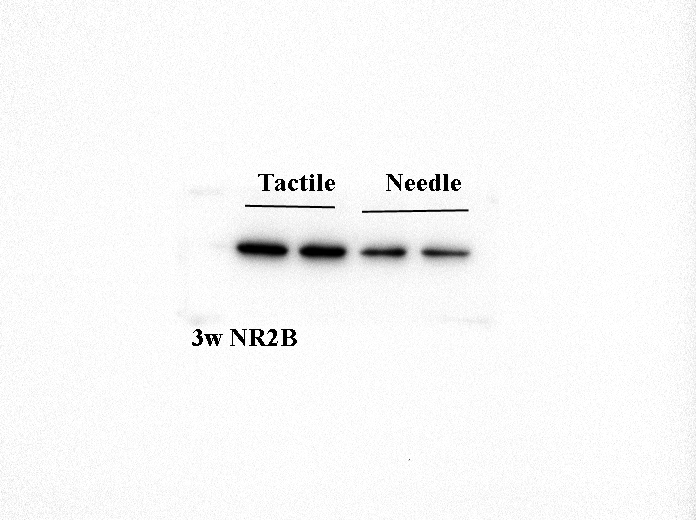

Supplement: Supplementary file 7 [file Data_Sheet_2.ZIP › 3W/3W,NR2B-3.jpg]
