# Supplementary material for: Repetitive Pain in Neonatal Male Rats Impairs Hippocampus-Dependent Fear Memory Later in Life
Source: Front Neurosci. 2020 Jul 8;14:722. doi: 10.3389/fnins.2020.00722 (PMC7360690; doi:10.3389/fnins.2020.00722)
Supplement: Supplementary file 5 [file Table_1.docx]

**Supplementary Methods**

**Measurement of mechanical withdrawal thresholds**

To assess whether the mechanical withdrawal thresholds (MWT) had changed after different neonatal treatments, the von Frey test was conducted using a set of calibrated filaments with a range from 2.83 (0.07 g) to 6.65 (300 g) (Stoelting Co, USA) on P22. Before measuring, the rats were placed individually on a wire mesh platform covered with a transparent acrylic cage for a 30-min acclimatization. The paw withdrawal thresholds were determined by the up-down method (Chaplan et al., 1994;Chen et al., 2016). Briefly, the calibrated von Frey filaments, starting from 4.56 (4 g), was applied to the plantar surfaces of the hind paws of rats. The MWT was defined as the lowest force to evoke positive nociceptive behavior (such as a brisk withdrawal of the paw, an attack or an escape reaction) in at least 3 out of 5 trials (>50%). With an interval of 5 minutes between each trial, sensitization could be effectively avoided.

**Supplementary Figure legends**

**Supplementary Figure 1.** Number of rats used in the preliminary (A) and Formal (B) experiment**.** FC: fear conditioning

**Supplementary Figure 2.** The MWT of the left hindpaw (A) and right hindpaw (B) on P23. Data are expressed as the mean± SEM. One-way ANOVA with the Bonferroni post-hoc test was used to compare the difference among the three groups. The MWT in the untreated group showed no difference with that in the tactile group, while the needle group showed reduced MWT of the bilateral hindpaws (Left hindpaw: F=9.230, P=0.001; Right hindpaw: F=16.022, P<0.001. ^a^P<0.001 for Needle vs. Untreated; ^b^P<0.001 for Needle vs. Tactile; ns, not significant; n=10 per group.

**Supplementary Figure 3.** Freezing behavior in Trace fear conditioning test in the preliminary experiment on P24-26. Data are expressed as the mean± SEM. (A) Freezing in the training phase. A repeated-measures ANOVA model showed no group difference in the training session (F=0.797, P=0.461). (B) Freezing in the contextual FC phase. In the training chamber, the Needle group showed reduced freezing time, but no difference was observed between the untreated and tactile group (P=1.000). In the novel chamber, no group difference was shown among three groups (F=0.081, P=0.923). (C) Freezing in the trace FC phase. A repeated-measures ANOVA model was used to analyze the group difference (F=4.54, P=0.02) and the pairwise comparisons at each time point was determined by the Bonferroni post-hoc test. The Tactile group behaved in the same pattern as the untreated group (P>0.05). ^a^P<0.001 for Needle vs. Untreated; ^b^P<0.001 for Needle vs. Tactile, n=10 per group.

**Supplementary Figure 4.** Expression of Synapse-related mRNAs (A-D) and proteins (E and F) in hippocampal. Data are expressed as the mean± SEM. The differences between the three groups were done by ANOVA and the pairwise comparisons were done by the Bonferroni post-hoc test. The NR1 (A), NR2A (B), NR2B (C), GluR1 (D) mRNA levels were presented as the ratio of each group to the untreated group, normalized to the corresponding GAPDH mRNA levels using the 2^−∆∆Ct^ method (n=10). NR1: F=6.839, P=0.004; NR2A: F=7.055, P=0.003; NR2B: F=7.659, P=0.002; GluR1: F=6.009, P=0.007, n=10 per group. The protein expression was presented as relative density (F) normalized to Actin, which was determined by densitometry analysis. NR1: F=6.304, P=0.01; NR2A: F=4.210, P=0.035; NR2B: F=6.946, P=0.007; GluR1: F=4.299, P=0.033. ^a^P<0.001 for Needle vs. Untreated; ^b^P<0.001 for Needle vs. Tactile, ns, not significant, n=6 per group.

**Supplementary Table 1. Body weight gain during the studying period**

| Age | Untreated (g) | Tactile (g) | Needle (g) |
| --- | --- | --- | --- |
| P0 | 5.92±0.08 | 6.06±0.07 | 6.08±0.09 |
| P8 | 19.25±0.25 | 19.39±0.23 | 15.21±0.64^a,b^ |
| P15 | 32.88±0.24 | 33.97±0.58 | 31.33±1.03^b^ |
| P22 | 55.69±0.51 | 55.04±1.15 | 55.63±1.06 |

Data were expressed as the mean± SEM. Repeated-measures ANOVA was used to compare main effects: F(age) =3293.99, P<0.001, and group F(group)=8.74, P=0.001. The pairwise comparisons at each time point were done using Bonferroni post-hoc test. ^a^P< 0.05 for Needle vs. Untreated, and ^b^P<0.05 for Needle vs. Tactile.
